# Expanding Community Health Worker decision space: learning from a Participatory Action Research training intervention in a rural South African district

**DOI:** 10.1186/s12960-023-00853-1

**Published:** 2023-08-18

**Authors:** Lucia D’Ambruoso, Nana Akua Abruquah, Denny Mabetha, Maria van der Merwe, Gerhard Goosen, Jerry Sigudla, Sophie Witter, the Verbal Autopsy with Participatory Action Research (VAPAR)/Wits/Mpumalanga Department of Health Learning Platform

**Affiliations:** 1 Aberdeen Centre for Health Data Science, School of Medicine, Medical Sciences and Nutrition, and Centre for Global Development, School of Education, University of Aberdeen, Aberdeen, Scotland, UK; 2 MRC/Wits Rural Public Health and Health Transitions Research Unit (Agincourt), School of Public Health, University of the Witwatersrand, Johannesburg, South Africa; 3 Department of Epidemiology and Global Health, Umeå University, Umeå, Sweden; 4 Public Health, National Health Service (NHS) Grampian, Aberdeen, Scotland, UK; 5 Department of Global Health, Stellenbosch University, Stellenbosch, South Africa; 6 The University Hospital, Kwame Nkrumah University of Science and Technology, Kumasi, Ghana; 7 Cochrane South Africa, South African Medical Research Council (MRC), Cape Town, South Africa; 8 Maria Van Der Merwe Consulting, White River, South Africa; 9 Mpumalanga Department of Health, Mbombela, South Africa; 10 Institute for Global Health and Development, Queen Margaret University, Musselburgh, Scotland, UK

**Keywords:** Community Health Workers, Participatory Action Research, Decision space, South Africa

## Abstract

**Background:**

While integral to decentralising health reforms, Community Health Workers (CHWs) in South Africa experience many challenges. During COVID-19, CHW roles changed rapidly, shifting from communities to clinics. In the contexts of new roles and re-engineered primary healthcare (PHC), the objectives were to: (a) implement a training intervention to support local decision-making capability of CHWs; and (b) assess learning and impacts from the perspectives of CHWs.

**Methods:**

CHWs from three rural villages (*n* = 9) were trained in rapid Participatory Action Research (PAR) with peers and community stakeholders (*n* = 33). Training equipped CHWs with tools and techniques to convene community groups, raise and/or respond to local health concerns, understand concerns from different perspectives, and facilitate action in communities and public services. CHWs’ perspectives before and after the intervention were gained through semi-structured interviews. Data were collected and analysed using the decision space framework to understand local actors’ power to affect devolved decision-making.

**Results:**

CHWs demonstrated significant resilience and commitment in the face of COVID-19. They experienced multiple, intersecting challenges including: limited financial, logistical and health systems support, poor role clarity, precarious employment, low and no pay, unstable organisational capacity, fragile accountability mechanisms and belittling treatment in clinics. Together, these restricted decision space and were seen to reflect a low valuing of the cadre in the system. CHWs saw the training as a welcome opportunity to assert themselves as a recognised cadre. Regular, spaces for dialogue and mutual learning supported CHWs to gain tools and skills to rework their agency in more empowered ways. The training improved management capacity, capabilities for dialogue, which expanded role clarity, and strengthened community mobilisation, facilitation and analysis skills. Development of public speaking skills was especially valued. CHWs reported an overall ‘tripe-benefit’ from the training: community-acceptance; peer support; and dialogue with and recognition by the system. The training intervention was recommended for scale-up by the health authority as an implementation support strategy for PHC.

**Conclusions:**

Lack of recognition of CHWs is coupled with limited opportunities for communication and trust-building. The training supported CHWs to find and amplify their voices in strategic partnerships, and helped build functionality for local decision-making.

**Supplementary Information:**

The online version contains supplementary material available at 10.1186/s12960-023-00853-1.

## Introduction

Community Health Workers (CHWs) in low- and middle-income countries (LMICs) play essential roles in primary health care (PHC) to achieve Universal Health Coverage (UHC) [1, 2]. Referring to all community/lay workers in the health sector, CHWs connect communities and health systems, through a range of functions including counselling and testing, education, follow-up and psychosocial support [3–5]. While supported in policy, CHWs face systemic biases and challenges in practice; limiting their potential and the anticipated benefits of community health action [2, 6, 7]. This article reports on a training intervention to support CHWs in their roles as they faced a new public health emergency, COVID-19, in rural South Africa.

CHWs have long been a part of the South African health system, albeit with variable recognition and support. CHWs were first trained as malaria assistants in the 1920s [8]. By the 1940s, despite growing segregation, social medicine advocates identified the need for integrated healthcare. This led to establishment of health centres, codified in the 1942 National Health Service Commission and the 1945 Gluckman Report [8–10]. Health centres were racially biased; however, only 10% catered to black communities [8, 9]. Government support waned, and many closed [9, 11]. There was a resurgence of CHW programmes in PHC in the 1970s; however, these collapsed with the National Health Plan, which discarded the cadre [2, 3].

Post-apartheid, the health sector transformed into a PHC system focused on equitable provision, prevention and health promotion [12]. There was a renewed focus on CHWs as task-shifting was adopted to manage the twin crises of HIV/AIDS and health worker shortages [13, 14]. Many CHW programmes were scaled as home-based care (HBC) was outsourced to community-based organisations (CBOs). CBOs employed CHWs on temporary contracts and paid them stipends to render services including: HIV counselling and testing; palliative care; Directly Observed Treatment (DOTs) for TB; and caring for orphaned and disenfranchised children. Although government supported the development of the cadre, low remuneration and poor working conditions continued [15]. By 2010, approximately 70,000 CHWs were employed by over 3000 CBOs across the country [16].

Decentralised health care is a long term health policy goal in South Africa; emphasising close-to-household care, health prevention, promotion, and community involvement [17–19]; *“empowering local administrative units to control their own healthcare agendas and resources…tailoring service provision to the local population* ([20], pp. 200). CHWs are a critical component decentralised health care, and their roles and functions are increasingly standardised as a result. In 2011, National Health Insurance (NHI) was introduced in a major shift towards UHC through removal of user fees. The main implementation strategy for NHI, ‘PHC re-engineering’ is organised around a national CHW programme: Ward-Based Primary Healthcare Outreach Teams (WBPHCOTs) [17].

WBPHCOTs comprise 6–10 CHWs per team, with nurse team leaders connected to local PHC facilities, catering for populations of approximately 7600 [16, 21]. The Department of Health (DoH) reports that WBPHCOTs have extended healthcare to homeless people and remote, marginalised populations, as well as to people lost to follow-up to increase successful treatment completion [21]. In 2017, a WBPHCOT policy framework was introduced to improve working conditions, recruitment, selection, placement, development, management, standardise workplans, ensure standard application and maintain and improve monitoring and evaluation [21]. Despite this, there remain low levels of integration of CHWs in the formal PHC system and WBPHCOT implementation has many challenges [16]. While a 12-month training curriculum was developed, funding is yet to be disbursed [22]. No clear leadership exists nationally, governance remains unclear, and investment is low [22]. CHWs are poorly remunerated and budget allocations are insufficient [22]. There is a disconnect in human resources management between nurse team leaders, employed and paid by the DoH, and CHWs who are paid (and sometimes not) by CBOs contracted by the DoH. More and proper allocation of resources have been called for to strengthen this stream of PHC re-engineering [16, 22].

CHWs played crucial roles in South Africa’s COVID response: delivering community and facility-based screening and testing, health education and behaviour change, case identification, referrals and linkage to facilities [23–25]. As first responders, CHW take up rapidly changing roles in pandemic responses [26]. Community awareness, engagement and sensitisation are, therefore, necessary, and CHW role clarity, training, supportive supervision, work satisfaction, health and well-being are critical [27]. Reflecting the systemic challenges for this cadre in the country, there were reports of CHWs at increased risk of infection and death owing to poor operational planning, lack of training, unavailability of Personal Protective Equipment (PPE), absence of incentives and lack of recognition [28, 29]. Calls for improved working conditions, including power to negotiate working conditions, were accompanied by industrial action and worker demands to be made permanent government employees, with better pay, medical aid, pensions, leave and sick pay instead of working under temporary contracts [29, 30].

There is unequivocal policy support for CHWs in South Africa, with major efforts to standardise their role and functions. Practical support to execute roles is limited, however. There is an urgent need for operational evidence on how accountability is transferred to lower levels in local clinics and hospitals, on how CHW programmes operate, and the impacts in rural areas, including power relations and personal change [18]. In 2020, a WHO review highlighted the need for more attention to: approaches to training; CHWs as social actors and community change agents; the influence of health systems decentralisation; and the voices of CHWs themselves [5].

The aim of this study was to assess, from the perspectives of CHWs, a community-based training intervention to support local decision-making during a public health emergency. The objectives were to deliver training to support functionality and local decision-making capability of CHWs in a rural sub-district, and assess impacts and learning from the perspectives of CHWs. Our focus was to understand whether and how the training intervention supported CHWs to undertake new and expanded roles during the COVID-19 pandemic, as well as within PHC reforms bringing services closer to people. The research questions were: (1) how do CHWs perceive and perform their roles in the context of PHC reform and COVID-19? and (2) how do CHWs assess the learning and outcomes of a training programme designed to support functionality through community mobilisation competencies in this context?

We used Bossert’s ‘decision-space’ framework of decentralisation in health system to understand local actors’ power to affect decision-making. The framework prescribes three dimensions. *Capacity* references resources and extent to which they are used, *authority* refers to clarity of roles and responsibilities, and *accountability* looks at mechanisms of responsibility within and outside the health system [31, 32]. Roman et al. expanded on this framework, identifying organisational capacity, resources, accountability mechanisms and the contexts in which these operate as synergistically enabling or constraining decision space in decentralised organisations [33]. The analysis adopted the Bossert and Roman frameworks to examine CHW perspectives on whether and how the training intervention supported them to undertake new and expanded roles during the first year of COVID-19.

The study was nested within the Verbal Autopsy with Participatory Action Research (VAPAR) programme. As a partnership between MRC/Wits-Agincourt and Mpumalanga DoH, VAPAR combines Verbal Autopsy (VA), a method to determine levels and causes of death in settings, where deaths go unrecorded, and Participatory Action Research (PAR), a process in which different stakeholders organise evidence for action (https://www.vapar.org/). The approach was rooted in health policy and systems research (HPSR), and constructivist and participatory paradigms. These assert that practical, experiential knowledge that is co-constructed, self-reflective, and embedded in complex, adaptive social and health systems can support and inform the organisation and delivery of equity-oriented and people-centred public services [34].

The VAPAR project consisted of three reiterative cycles of collective data gathering, analysis, action and reflection originally in three villages with different levels of socioeconomic status and access to services [35]. Local health concerns were raised and framed by around 50 community stakeholders across the three villages, who generated new data and evidence on these using VA and PAR. Following community-based problem analyses and development of action agendas, dialogue was developed with local authorities. A further 15–25 government department and NGO actors then joined ‘learning platform’ workshops with community stakeholders and researchers, where data were presented and appraised, and local action plans were jointly developed, implemented, and monitored. Finally, we collectively reflected on and reiterated the process. The first cycle initiated collective capabilities, and data and evidence were built and acted on by community stakeholders and the authorities [36–38]. Responding to COVID-19, the second cycle was interrupted and re-designed to include a training package for CHWs in rapid PAR [39]. The training was rolled out in the third cycle, in which the current study was nested.

## Methods

### Study setting

The project was based on the MRC/Wits Agincourt Health and Socio-Demographic Surveillance System (HDSS) in Mpumalanga, a rural province of 4.7 million. Agincourt HDSS is among Southern Africa’s oldest and largest population-based cohorts, covering a population of 120,000 in 31 villages over 450km2. Village populations vary from < 5000 to > 10,000. Former Mozambican refugees and their descendants comprise about 30% of the population, and there are many orphaned youth: school drop-out is 40%, 16% of the provincial population is illiterate, and district unemployment is 37% [40]. The rural sub-district is served by two community health centres and seven PHC clinics [41]. Agincourt’s 30-year longitudinal surveillance reveals a prolonged epidemiological transition. Multiple burdens of death and disability; HIV/AIDS, non-communicable diseases, maternal and perinatal mortality and injuries, underscore an acute need for integrated planning and service delivery [40].

### Participant recruitment and data collection

As COVID took hold in early 2020, we adapted the process to provide practical support to the district health system. A rapid, largely virtual consultation with community stakeholders, government officials and multisectoral resilience fora revealed that CHWs were seen as first-line responders, but needed support to meaningfully engage with communities. The third action-learning cycle was, therefore, co-redesigned to support CHWs to develop community mobilisation competencies through training in ‘rapid PAR’ to: (1) build capacities in rapid evidence generation on local health issues; (2) support utilisation of evidence in district planning and management, specifically PHC planning and review; and (3) further develop collective action supporting community responses addressing social determinants [39]. (N.B.: this paper reports on implementing the training intervention, and assessing learning and impacts from the perspectives of CHWs, which are not the same as the objectives of the third VAPAR cycle).

In November 2020, we reviewed a training framework and accompanying manual with operational managers (OMs) from clinics serving the study villages to ensure local relevance and practical utility. Invitations were then extended to CHWs through OMs to enrol in the 10-week certified training programme. OMs and researchers selected three CHWs from each clinic catchment area based on interest, skills, and motivation (*n* = 9 in total). Community stakeholders involved in the previous cycles and conversant with the process [42] joined the process as ‘community mentors’ supporting CHWs with peer-learning and exchange (*n* = 6 in total).

The training programme was then rolled out (Table 1). CHWs, mentors, PHC staff, OMs, and clinic committee members from the three clinics attended an initial workshop. Researchers introduced the training, emphasised participatory principles around democratic involvement free of blame, shared ownership and action. Participants discussed local health concerns, which were listed to capture different perspectives and experiences. Participants then ranked the lists, agreeing on priority issues to focus the training around. Two further workshops were held with CHWs and community mentors to orient them to the PAR tools to: elicit and systematise lived experience; collectively analyse and problematise; collect and interpret visual data; and reflect and learn. CHWs then recruited nine participants from each village (*n* = 27 in total). ‘Community stakeholders’ were recruited as individuals directly affected by the issues identified, and whose voices might be excluded.

**Table 1:** Rapid PAR training framework

Week/Workshop	Focus	Participants	Facilitator/s	Activities
Week 1 Workshop 1	Introduction Training	• CHWs [9] • Community mentors [6] • Clinic nurses [3] • Clinic committee [3]	Researchers	• Training in PAR tool 1 (listing/ranking/rating) to support and orient CHWs and community mentors to identify and prioritize focus health issue(s)
Weeks 2–3 Workshop 2	Training	• CHWs [9] • Community mentors [6]	Researchers	• Training in PAR tool 2 (problem tree) to identify priority health-related condition, facilitation and consensus building and community engagement—engaging active participant selection and representation (n = 3 for each CHW) • Training in PAR tool 3 (Photovoice) to convey lived experience visually. Basic photography training conveying topics in context and showcased in discourses. Captions on images • Baseline interviews
Week 4 Workshop 3–5	Problematising	• CHWs [3] • Community mentors [2] • Community pax. [9]	CHWs and community mentors	• Application of PAR tools 2 and 3 to understand priority health issue from different perspectives, identifying cause-and-effect relationships, consequences, and other effects, building shared accounts. Orient community pax. to collection of visual data
Week 5 Workshop 6	Training	• CHWs [9] • Community mentors [6]	Researchers	• Reflect on week 4 workshops [3–5] • Training in PAR tool 4 (Venn diagram) to identify actors and organisations involved with the topic and how they relate to one another with regard to collaboration and contact
Week 6 Workshop 7–9	Actors and impacts	• CHWs [3] • Community mentors [2] • Community pax. [9]	CHWs and community mentors	• Application of PAR tool 4 to understand actors and impacts. Collective account indicating relationships and interactions between various actors and institutions
Week 7 Workshop 10	Training	• CHWs [9] • Community mentors [6]	Researchers	• Reflect on week 6 workshops [7–9] • Training in PAR tool 5 (Action planning) to collectively articulate overall goal(s) and develop series of interconnected steps and events to achieve these
Week 8 Workshop 11–13	Action planning	• CHWs [3] • Community mentors [2] • Community pax. [9]	CHWs and community mentors	• Application of PAR tool 5 to agree overall goal(s) to address issues identified and develop stepwise actions to achieve these. Draw on problem tree and Venn diagram to collectively articulate a desired goal and a series of interconnected steps and events
Week 9 Workshop 14	Synthesis and process for engaging authorities	• CHWs [9] • Community mentors [6] • Clinic nurses [3] • Clinic committee [3]	Researchers	• Reflect on week 8 workshops [11–13] • Codesign/plan taking local action plans to multisectoral fora for analysis and planning • End-line interviews
Week 10 Workshop 15	Reflect on evidence, planning engagement	• CHWs [9] • Community mentors [6] • Clinic nurses [3] and • Clinic committee [3] • Community pax. [27]	CHWs and community mentors	• Present and finalise PAR data for 3 villages • End-line interviews

N.B.: shaded rows indicate community workshops held in each of 3 villages; clear rows denote training/peer support workshops. ‘Pax.’ denotes participants

Weekly workshops followed in each area. Workshops were alternated between ‘practice-based’ CHW/mentor-led sessions applying PAR tools with community stakeholders, and ‘theory-based’ researcher-led training sessions with CHWs and mentors only. The latter were to; reflect on use of PAR in community workshops, and build experience with peer-support. Throughout, PAR tools were sequenced to problematise local health concerns, reflect on, and appraise action. Workshops were held in churches, community centres and other community spaces in local languages siTsonga and Shanga’an, and mentors adopted roles as co-facilitators with CHWs. In all workshops, CHWs, mentors and researchers worked to facilitate safe spaces free of blame and promoting democratic involvement.

A final workshop convened health system and community stakeholders to synthesise findings, and codesign subsequent multisectoral engagement with local officials and non-governmental agencies. Community stakeholders also collected and interrogated visual evidence using Photovoice, whereby participants collected images of local environments, and collectively selected and appraised visual evidence as part of the weekly workshops. VA data were employed as a further input to the workshops, quantifying disease burdens, levels, medical causes, and social and health systems determinants associated with community-nominated priorities. Throughout, relevance to and incentives and motivations among CHWs was a key focus to ensure that the process was a feasible and realistic addition to CHW activities.

The pre–post evaluation assessed learning and impacts from the perspectives of CHWs. The researchers conducted baseline interviews with CHWs. These were semi-structured using the decision space framework to explore CHWs’ power to act at community, facility, and district/sub-district levels. End-line interviews were also structured by the framework, focussing on whether and how the training intervention influenced decision-space and reflecting on learning and impacts. Interviews were 30–60 min long, conducted in the local language, siTsonga by siTsonga-speaking researchers, and structured by pre-prepared topic guides. Data were anonymised, transcribed, and translated to English, and quality checks performed. Audio recordings were reconciled with transcriptions and a set of transcripts were back translated. Data were encrypted and stored on institutional servers in MS Word, and other project data were stored in MS PowerPoint, and image files. There were no refusals in recruitment of CHWs, community mentors and community stakeholders and no dropouts.

### Analysis

The analysis sought to assess, from the perspectives of CHWs involved, whether, how and where the training intervention supported functionality for local decision-making, in the contexts of COVID-19 and decentralising PHC. The focus on CHWs responds to calls for evidence on the voice of CHWs [5] and is intended to complement evaluations from the perspectives of other key stakeholders [38, 43, 44]. As described above, we adopted Bossert’s framework on decentralisation in healthcare to explore local actors’ power to affect decision-making [31, 32], with Roman et al.’s interactive extension in which the elements work synergistically to enable or constrain decision space [33]. Interview data were the main data source, supplemented by presentations, registers, minutes, fieldnotes, and researcher exchanges*.* Thematic analysis was performed using NVivo 11 and 12 to identify inductive and deductive themes. Codes were arranged into themes and sub-themes and were arranged by the analytical framework.

### Ethical considerations

Written consent was gained from all participants. Participants were provided with written and verbal information on the study and provided minimum 72 h for questions to be asked and answered. Anonymity was ensured as was freedom to exit the process at any time and for any reason including no reason. Participants were provided with refreshments and transport costs for workshops, and reimbursed for time participating, 300ZAR (approx. 15GBP) per participant for the 10-week training module. All identifiable data were anonymised. Electronic data were stored in password-protected files on secure servers hosted by Agincourt HDSS and University of Aberdeen. The study was approved by Mpumalanga Health Research Committee (MP_201712_003). Protocols were approved by the Research Ethics Committees of University of the Witwatersrand, Johannesburg (M171050), and University of Aberdeen (CERB/2017/4/1457).

## Results

Below, we present the analysis of CHW perspectives regarding systems constraints and barriers to local functionality (pre-interviews) and the mechanisms through which the training intervention worked to enable impacts for local decision-making (post-interviews). The thematic analysis is presented below, illustrated with verbatim quotes, and in Tables 2, 3, and 4.

**Table 2:** Thematic analysis on ‘Capacity’ domain: resources and capacity to use them

(i) Pre-intervention		(ii) Post-intervention	
Sub-themes	Quotes (barriers)	Sub-themes	Quotes (enablers, impacts)
Barriers • Lack of training	“For someone who had never worked at the clinic before, I wish I had proper training … That is what they promised us when we got to the clinic, but they didn’t deliver, we are even promised a full COVID-19 training but we are still waiting.” Participant, clinic 1	Enablers • Structured/certified process by Wits/DoH • Co-designed process: practical, acceptable and locally relevant • Inclusive ‘safe to fail’ learning spaces • Principles of collective knowledge and action regularly revisited and appraised	“The VAPAR training has filled a lot of gaps that I had before I joined the training. The first one is public speaking, I was so shy when I joined but the training helped me overcome the challenge, now I can talk in front of people”. Participant, clinic 1
	“I do not have challenges at the community but at the clinic, I feel like the clinic does not care about us. They do not care about our training needs, salaries, and other things. I wish they can start to recognise us more and treat us like we are also professional health workers.” Participant, clinic 1		
	“I need training on report writing because I still have challenges with it.” Participant, clinic 3		“The VAPAR training helped me with ways of prioritising, …This will often create tension because each one of us want to be heard but now I have learnt about … systems that allow everyone to have an equal voice and enable us to reach an agreement without undermining each other.” Participant, clinic 1
	“We want to be trained so that we qualify as assistant nurse.” Participant, clinic 2	Impacts • Improved communication and mutual respect (CHWs, DHS actors and communities) • Collective mindsets built • Competencies built in community mobilisation • Competencies built in public speaking • Competencies built in data analysis • Competencies built in peer relationships • Competencies built in alliance building	“I will use the facilitation skills I obtained during the training when we have meetings at the clinic and when conducting support groups.” Participant, clinic 3
Barriers • Insufficient equipment and supplies	“We do not have enough equipment; most challenging now is inadequate transportation, we either have to walk very long-distance crossing rivers to access our patients or even use our own money to pay for transport, and this affects our work negatively.” Participant, clinic 3	Enablers • Platform enabled logistical challenges to be referred to OMs/systems actors	“The training taught me ways of identifying challenges and addressing them, I understand challenges better than I used to. I'm confident that now I know even how to identify people who can assist us in dealing with various issues” Participant, Clinic 1
Barriers • Inadequate remuneration	“We are still waiting about the COVID money that they promised to pay us when we were done with COVID-19 screening…. Now they made us risk our lives to do registrations for COVID-19 vaccines, but they are not going to pay us at the end.” Participant, clinic 3	Enablers • Reimbursement for time in training • Forum for dialogue on pay/conditions Impacts • Requests for more training programmes	“DoH can support by increasing our salaries and for VAPAR to continue giving us training.” Participant, clinic 3
	“My wish is for the government to recognise our value and the effort we put in saving the lives of people in our communities” Participant, clinic 1		
Barriers • Poor employment and career development	“It is frustrating to see our community suffering while we know we can help them, but we cannot because we are not allowed to work.” Participant, clinic 2	Enablers • Forum for dialogue on employment and career development	“I know that VAPAR does not have the capacity to employ us, but if they can motivate for us or negotiate with DoH on our behalf we would really appreciate.” Participant, clinic 2
			“Maybe VAPAR can convince the department on our behalf, I desperately need to go back to work because I feel like my patients will default their medications because they have no one to help them.” Participant, clinic 2

**Table 3:** Thematic analysis on ‘Authority’: clarity in roles and responsibilities

Pre-intervention		Post intervention	
Sub-themes	Quotes (barriers)	Sub-themes	Quotes (enablers, impacts)
Barriers • Hierarchical organisational culture punitive and disrespectful towards CHWs • Initial concerns that training intervention punitive and designed to ‘expose weaknesses’	“What I learnt about the clinic system and at the clinic is that there is a lot of discrimination, especially for CHWs like us who have not been trained by WBPHCOT.” Participant, clinic 1	Enablers • Engaging in respectful dialogue • Stable platform • Peer modality • Process framed for respect and development of shared mindsets • Enjoyable process of co-learning and action	“The training was challenging in the beginning, maybe it was my attitude towards it but is the training continued I started enjoying it. I had a lot of fun engaging with others, especially the clinic managers, on days that they attended, it felt like the managers are paying attention and are suddenly interested in what CHWs do. I learnt a lot from the training.” Participant, clinic 3
			“…the training also helped us to engage better with the nurses and to realise our relevance in both the community and the health facilities.” Participant, clinic 3
	“… we don’t feel welcome at the clinics because of how we are treated”. Participant, clinic 3		“At first I was shy to facilitate or to even raise my opinion, but I later adapted because while you were training us you made us feel equal and you always showed us respect.” Participant, clinic 1
	“We do have support from the clinic although there are some nurses who still have bad attitude toward us such that they either don’t have time to assist when we need help, or they are not patient with us.” Participant, clinic 3		“The VAPAR training addressed a lot of things that I didn’t know for example, as CHWs we are expected to conduct health talks, but it was challenging for me because I am a shy person but after attending the training, I learnt a lot about public speaking and ways of engaging with other people.” Participant, clinic 1
	“I would say, generally, we get enough support at the clinic … however I wish all the nurses at the clinics could recognise us as their colleagues. Sometimes we don’t feel welcome at the clinics because of how we are treated” Participant, clinic 3	Impacts • Communication skills built to engage supervisors • Negotiation skills built • Trust relationships strengthened, and quality relationships built • New collaborations to work towards shared goals	“My experience with the training was good. At the beginning I thought that you wanted to expose when you asked us to facilitate the workshops but as time goes on, I realised that you were training us to be better CHWs because now I can speak confidently and my report writing have improved” Participant, clinic 1
Barriers • Knowledge on CHW roles mixed	“The roles are not clear because we are stationed at different working stations, there is no rotation and there are no clear criteria as to why certain people are stationed at certain stations.” Participant clinic 1	Enablers • Dialogue and awareness raising regarding challenges owing to lack of role clarity	“VAPAR training addressed a lot of things that I didn’t know for example, as CHWs we are expected to conduct health talks, but it was challenging for me because I am a shy person but after attending the training I learnt a lot about public speaking and way of engaging with other people.” Participant, clinic 1
	“My roles and responsibilities were clear… I know what I need to do at the community, and I also know what I need to do at the clinic.” Participant, clinic 2		“The training highlighted some of our responsibilities as CHWs, but I am still not certain about what is expected of me at the clinic.” Participant clinic 1
	“I will not say everyone in the community understands what we do because there is still a lot of confusion… people still don’t believe that we are trained to support them with getting their chronic medication which is why we have a lot of defaulters.” Participant, clinic 3	Impacts • Role clarity improved • Competencies built in community mobilisation • Competencies built in public speaking • Competencies built in peer relationships	“…the VAPAR training helped us understand some of our responsibilities. It made me understand my role as a CHW because as we met with other CHWs from other clinics, we got to learn exactly what is expected of us” Participant, clinic 1
Barriers • Workloads high, pay and conditions insufficient • ‘Double burden’ of clinic and community roles	“…lately targets don’t matter because we neglect some of our responsibilities to assist the clinic whenever they need us.” Participant, clinic 3	Enablers • Resilience and commitment of CHWs to community health deep and enduring • Forum to broker dialogue on workloads with OMs/higher systems actors	“…now I have a better understanding of the role of different stakeholders at the clinic and how effective us as CHW can benefit from working with them.” Participant clinic 1
	“I go to outreach and come back to work at the clinic, sometimes I spend the whole night compiling reports, the other time I had 50 people that I needed to capture, and they expected me to submit the report the following day.” Participant, clinic 3

**Table 4:** Thematic analysis on Domain 3: accountability: mechanisms of responsibility within and outside the health system

Pre-intervention		Post-intervention	
Sub-themes	Quotes (barriers)	Sub-themes	Quotes (enablers, impacts)
Barriers • Community accountability generally good, reports of trust mixed	“The community still gives us support; however, we are not in contact with them like we used to since now we spend a lot of our time working at the clinic.” Participant, clinic 3	Enablers • Stable platform for building trust and action alliances in response to local needs, e.g., patient support groups dealing with stigma/loss to follow-up • Process to convene and mobilise community stakeholders to raise awareness and address stigma over COVID-19 and other communicable diseases • Better quality relationships between CHWs	“The VAPAR training was good; it met my expectations. I learned a lot about respect, communication and how to use all the tools … Most importantly I learnt the power of working together as CHWs, communities and traditional authorities. If the communities can master this approach of working together, we can solve a lot of issues that our communities come across every day.” Participant clinic 2
			“Personally, I had challenges with public speaking but since I attended the training my presentation skills have improved. Now I can confidently facilitate any workshop at any given time. I learned from how you were engaging with us, it was the first time we met but you made us feel free to talk around you and that is something I want to practice with my patients in the community, making people feel free and welcomed.” Participant, clinic 3
			“The training exceeded my expectation; I learnt a lot more than I expected …. I will use the skills I leant during the training to identify issues faced by chronic patients and work with them to address these issues.” Participant, clinic 1
	“Our relationship is good with the community; they are starting to see the importance of our work. Others will also tell you they miss seeing us in the village, when they see us they get very happy.” Participant, clinic 1		“I will use a problem tree to help my patient understand their medical condition better, to understand the cause of the complications and the impact of not adhering to their medications.” Participant, clinic 2
	“Our relationship [with the community] is well except that other people still don’t know what our roles are in the community, as a result some are still reluctant to our services… Communities need to be educated more about our roles” Participant, clinic 3	Impacts • Strengthened trust relationships between CHWs and communities • Development of shared mindsets	“The training … helped me to understand public engagement more, how to be patient when working with the local people and to understand deeper issues around defaulting of HIV/TB.” Participant, clinic 3
Barriers • Supervision generally adequate	“I currently report to sister [name], we have a good working relationship where we can ask anything anytime, we want. She gives us feedback in a professional manner.” Participant, clinic 1	Enablers • Increased understanding of alliance-building and collaboration	“VAPAR training played a huge role to me as an individual because I did not know how to approach problems that we encounter as Community Health Workers but now I know. The training taught me ways of identifying challenges and addressing them, I understand challenges better than I used to. I'm confident that now I know even how to identify people who can assist us in dealing with various issues.” Participant, clinic 2
	“I hardly get feedback or communication regarding my performance; I think it's because [clinic name] has a lot of CHWs so it’s difficult to keep track of each of us.” Participant, clinic 1		“…my confidence and communication skills have improved. I am communication better with the nurses and community people. My thinking has improved because I know how to identify problems and finding solutions for them.” Participant, clinic 3
	“We used to get proper supervision where we're still allowed to work but not anymore, since we were told to stop working. Suddenly, even the nurses do not care about our existence. Now, no communication at all such that we do not even know whether we are needed at the clinic or not but back then they used to communicate very well with us.” Participant, clinic 2	Impacts • Opportunities to build dialogue at higher levels of system, supporting more effective relationships including supervision	“The VAPAR training exposed me to things that I didn’t know such as identifying various problem within our community and how we can collectively solve them. I learned that before one can start thinking about solution of a problem, they should start by understanding the problem, what are the causes of these problems and its impact. I have also learned about how we can identify stakeholders at different levels to engage with them in solving community issues.” Participant, clinic 2

### Capacity: resources and capacity to use them

All respondents articulated resourcing challenges. Training was seen as a major problem. Most CHWs were certified carers; however, few were WBPHCOT-trained. CHWs recounted how they had urgently needed, and had been assured, training including for new duties related to COVID-19. Promises of training were unfulfilled, however. Insufficient training was seen to reflect CHWs’ low value to in the system and as limiting of future career prospects, including through pathways to nursing. In this scenario, CHWs reflected that the training was a welcome opportunity to assert themselves as a recognised cadre.

Some CHWs initially felt the PAR tools were challenging. Confidence developed, however, through weekly meetings, framed as ‘safe-to-fail’ spaces with regular revisiting and appraising of principles of democratic involvement free of blame, shared ownership, and action. CHWs recounted that the weekly encounters supported them to build completely new skills and competencies in communication, community mobilisation, public speaking, data analysis, peer relationships and alliance building. Public speaking in particular was seen as an important gap in professional competencies that was filled by the training The general sentiment was that more training was required and continued training was requested.

“The VAPAR training has filled a lot of gaps that I had before I joined the training. The first one is public speaking, I was so shy when I joined but the training helped me overcome the challenge, now I can talk in front of people”. Participant, clinic 1

“The VAPAR training helped me with ways of prioritising …This will often create tension because each one of us want to be heard but now I have learnt … systems that allow everyone to have an equal voice and enable us to reach an agreement without undermining each other.” Participant, clinic 1

Otherwise in terms of capacity, CHWs recounted acute logistical challenges that were exacerbated by the pivot to clinic-based responsibilities. There were shortages of PPE, unavailable transport, and missing equipment. CHWs reported frequent use of their own resources to fill gaps. CHWs also reported inadequate pay. Most CHWs were DoH-funded and working at facilities. However, some still operated under CBOs that were unfunded, and non-receipt of stipends were recounted. In some cases, payment had not been received since 2015. Other CHWs engaged by clinics had yet to receive remittances for COVID-19 screening. For those who received remuneration, it was seen as insubstantial as a living wage. CHWs recounted how they remained committed; however, lack of fair pay was seen as further evidence of low recognition and value by the system. The training intervention provided stipends, travel costs and refreshments. While this was not a solution to insufficient equipment and pay, the skills and competencies that CHWs built during the training supported them to broker dialogue with the DoH and other actors to refer and resolve broader systems challenges.

“The training taught me ways of identifying challenges and addressing them, I understand challenges better than I used to. I'm confident that now I know even how to identify people who can assist us in dealing with various issues” Participant, Clinic 1

CHW narratives reflected fundamental issues of employment as a permanent, central feature in PHC. All participants had been working at post for a minimum of 5 years and had well-developed skills in community-based care including complex care for chronic multimorbidity and ageing. CHWs not employed by the government felt that recognition would increase their capacity and effectiveness, especially in the context of the pandemic, and several expressed frustration and concern; feeling helpless to attend to unmet needs in communities. The training intervention had no influence employment, other than CHWs reported developing new skills to engage in constructive dialogue and action on government contracts and career development. Requests were made for VAPAR to broker these discussions.

“I know that VAPAR does not have the capacity to employ us, but if they can motivate for us or negotiate with DoH on our behalf we would really appreciate.” Participant, clinic 2

### Authority: clarity in roles/responsibilities

CHW’s authority was deeply undermined by a pronounced lack of role clarity, since the expansion of duties into clinics. CHWs also described belittling and unfair treatment from colleagues in clinics. WBPHCOT-trained CHWs narrated disrespect from fellow health workers and untrained CHWs experienced blatant discrimination. Unfair treatment of CHWs in clinics transmitted further messages regarding low value and recognition in the system.

CHWs reported initial anxiety about the training and concerns that their ‘weaknesses’ would be exposed. As the process unfolded, however, they reported, gaining confidence, and new forms of authority through a process grounded in principles of mutual learning and respect. The peer-led spaces aided in bonding, helping CHWs to recognise one another, as well as raise a collective voice to address challenges with colleagues at higher levels. Through the peer modality, quality relationships between CHWs were seen as a positive experience and strategic benefit; and CHWs described the process a positive and ‘fun’ activity. CHWs also reported learning how to communicate more effectively with supervisors and communities prioritising mutual respect and providing new opportunities for senior colleagues to appreciate local health concerns at a deeper level and from different perspectives. Overall, these served to provide a ‘triple-benefit’, addressing broader systems challenges via community-acceptance; peer support; and dialogue with and recognition by the system.

“The training was challenging in the beginning… as the training continued … I had a lot of fun engaging with others, especially the clinic managers, on days that they attended, it felt like the managers are paying attention and are suddenly interested in what CHWs do. I learnt a lot from the training.” Participant, clinic 3

“…the training also helped us to engage better with the nurses and to realise our relevance in both the community and the health facilities.” Participant, clinic 3

“At first I was shy to facilitate or to even raise my opinion, but I later adapted because while you were training us you made us feel equal and you always showed us respect.” Participant, clinic 1

CHWs were knowledgeable about roles and responsibilities, although ‘untrained’ (non-WBPHCOT trained) CHWs expressed uncertainty over what their roles would be in future. CHWs narrated that a major benefit from the training had been improved role clarity together with capacity building to fulfil better defined roles. This was achieved through the peer modality for support and exchange, and dialogue and development of shared mindsets with communities and in the health system.

“VAPAR training addressed a lot of things that I didn’t know for example, as CHWs we are expected to conduct health talks, but it was challenging for me because I am a shy person but after attending the training, I learnt a lot about public speaking and ways of engaging with other people.” Participant, clinic 1

“The training highlighted some of our responsibilities as CHWs, but I am still not certain about what is expected of me at the clinic.” Participant clinic 1

Workloads was a further theme that related to role clarity. CHWs recounted generally manageable workloads prior to the pandemic. However, recent demands had significantly increased workloads, with regular abandoning of targets. Some CHWs described a ‘double burden’ workload in communities and clinics. Despite these challenges, CHWs expressed deep commitment to render services in rural and underserved communities. While the training had modest impacts on workload, CHWs felt equipped with tools to broker dialogue in clinics and at higher levels.

“…now I have a better understanding of the role of different stakeholders at the clinic and how effective us as CHW can benefit from working with them.” Participant clinic 1

### Accountability: mechanisms of responsibility within and outside system

Accountability was generally narrated as high. As described above, CHWs asserted that the systemic challenges affected neither the services they rendered nor the supervisors and system they reported to. All CHWs felt they had overall good working relationships with communities and felt accountable for residents in their care. There were, however, some descriptions of detachment from previous roles owing to COVID-19, and some respondents expressed a sense of loss of contact with communities. Trust was a further theme. Some CHWs reported good levels of trust with communities after many years working in rural areas. Others experienced resistance despite efforts to engage. CHWs explained this was due to communities’ concerns and perceptions over lack of confidentiality over COVID-19 and other communicable diseases.

CHWs narrated how the training provided both a platform and a process to develop new alliances between communities and services, build trust and advance collective recognition of and action towards local concerns, such as stigma around communicable diseases [45]. This was achieved through workshops that provided bonding opportunities for participants and helped them to recognise each other as key agents in PHC provision. CHWs assessed the intervention to be important in helping to facilitate, convene and mobilise community members to raise awareness, de-stigmatise COVID-19 and other communicable diseases, as well as other health and community concerns including chronic illness management. The tools were seen to provide a mechanism to strengthen the ties that some CHWs already had with communities, and as offering new opportunities to form alliances with those who had challenges with trust.

“ learned a lot about respect, communication and how to use all the tools ... Most importantly I learnt the power of working together as CHWs, communities and traditional authorities. If the communities can master this approach of working together, we can solve a lot of issues that our communities come across every day.” Participant clinic 2

“I learned from how you were engaging with us… you made us feel free to talk … and that is something I want to practice with my patients in the community, making people feel free and welcomed.” Participant, clinic 3

“The training exceeded my expectation; I learnt a lot more than I expected …. I will use the skills I leant during the training to identify issues faced by chronic patients and work with them to address these issues.” Participant, clinic 1

All participants recounted knowledge of supervision structures in clinics as accountability mechanisms, and many felt supported by superiors. They did not always get feedback concerning performance, however, generally because of systems overload. CHWs reflected on benefits of the training in terms of new alliances, dialogue, and processes to build mutuality. Being able to address problems through relationships in clinics as well as communities was seen as a significant benefit and expanded confidence and skills.

“VAPAR training played a huge role to me as an individual because I did not know how to approach problems that we encounter as Community Health Workers but now I know.” Participant, clinic 2

“…my confidence and communication skills have improved. I am communication better with the nurses and community people. My thinking has improved because I know how to identify problems and find solutions for them.” Participant, clinic 3

During the final workshops, planners and managers acknowledged the value of CHWs and cooperative learning: “I never knew how much these people know…we never knew we could learn from these people” (System actor, reflection workshop report) and “For the first time, CHWs and managers sit at one table and engage” (System actor, reflection workshop report). There was a collective reflection on completion of the cycle, with CHWs, community stakeholders and health systems actors (clinic nurses, Outreach Team Leaders [OTLs], OMs and sub district PHC managers), which concluded with was a recommendation to scale the training outside the study setting (population 130,000) and across the sub-district (population 550,000) [46].

**Fig. 1:**
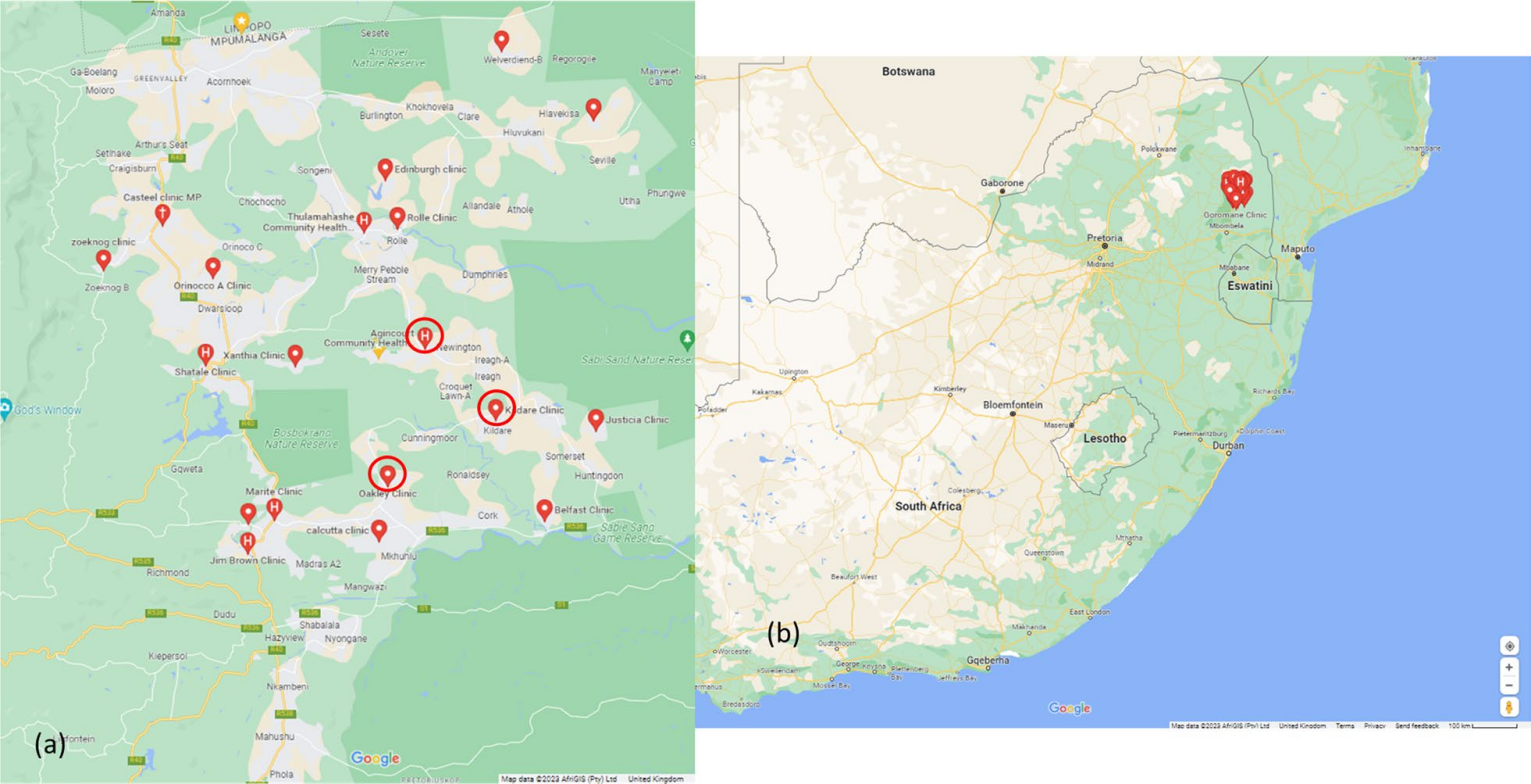
‘Cycles 4 and 5′: expansion of intervention across sub-district. **a** clinics in Cycle 3 (circled) and 4 and 5; **b** scaled map of location of training programme (image Credits: Hanna-Mari Van Der Merwe, Google Maps)

**Fig.2:**
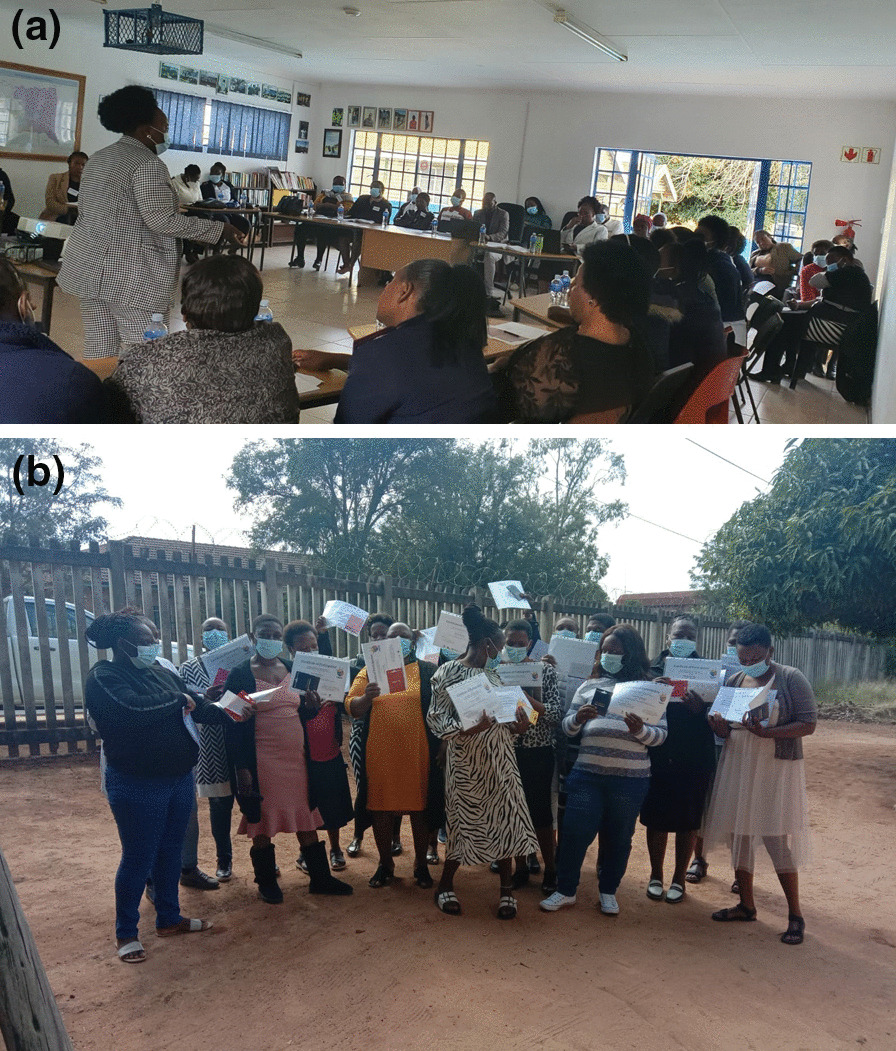
**a** Scale-up of training (image reproduced with permission). **b** Close-out celebration of training scale-up (image reproduced with permission)

Following codesign/reiteration the researchers mobilised resources to roll out a further cycle with fifty CHWs in six local areas across the sub-district (Fig. 1) [46]. The training manual was revised and provided as an open access resource, with versions in siTsonga and English, jointly produced with the DoH for use by CHWs working as part of WBPHCBOTs (Additional files 1, 2) [47, 48]. There was regular attendance at workshops by OTLs and clinic OMs during the scale-up, and health workers (Fig. 2a). CHWs again reported feeling more recognised by the health system, communities and local authorities; with improved engagement and partnership and recognition of skills and competencies (Fig. 2b). Sub-district management formally acknowledged CHW roles, confirming the urgent need to address CHWs’ employment status [49]. We also developed a radio series, broadcast on two local stations with programmes in siTsonga to amplify CHWs voices and inform communities of their practice and role as entry points to healthcare [50].

Following the scale-up, a sub-district ‘CHW platform’ was established by OTLs with CHW representatives from each local area in the sub-district and supported by the research team. Still operational at the time of writing, the CHW platform supports peer-to-peer support and exchange with clinic staff and sub-district management teams to share lived experiences from communities as observed and reported during community engagement activities [49]. Finally in 2023, and responding to a request from the Department, the team secured additional funding to: (a) develop a ‘Training-of-Trainers (ToT)’ package delivered to an additional 60 CHWs, together with health systems and community actors across the sub-district (population 550,000) and (b) evaluate with the DoH to explore effectiveness and leverage support for further uptake as an implementation support strategy for PHC Re-engineering [51].

## Discussion

This study assessed, from the perspectives of recipient CHWs, a training intervention to support professional functionality and local decision-making capability in contexts of decentralising PHC and a public health emergency. The pre–post evaluation surfaced multiple systems constraints that, together, significantly restricted CHW decision space; disconnecting people and services and reversing PHC policy goals. The systemic norms and biases constraining CHW decision space also transmitted clear signals to CHWs about their low value and worth to the system, and did so at a time when CHWs displayed extraordinary resilience and commitment in face of COVID-19.

In terms of capacity and resourcing, there was inadequate training, low and no pay, precarious employment, hazardous working conditions, unmanageable workloads, poor career progression, and critical equipment shortages. Training was a major issue. WBPHCOTs-trained CHWs had some advantage in clinic settings, while untrained and unemployed CHWs struggled to adapt and be recognised. In terms of authority, CHWs experienced discrimination and disrespect as they shifted to support clinics in COVID responses. There was poor role clarity and limited opportunities for communication and trust-building. While supervision structures were generally clear and relationships with communities overall good, accountability mechanisms were fragile.

The intervention contributed to CHW decision space in different ways and through different mechanisms. There were modest, defined improvements in roles (authority) and resources (capacity). Provision of vital training was well-received, as were capabilities for dialogue, which drove greater role clarity. Supportive learning spaces helped CHWs develop analytical, facilitation and public speaking skills, which built individual and collective agency. Public speaking skills were especially valued.

The intervention also improved connections (accountability). CHWs narrated the ‘triple-gain’ of building shared mindsets, trust and communication with communities, the health system, and through quality peer-to-peer relationships. CHWs reported that the principles of democratic involvement and respectful dialogue unlocked learning for them, as well as enabling peer learning and strategic alliance building. The platform closed at least some of the distance between CHWs and higher level systems actors. While employment, careers, pay and conditions were harder for the intervention to address, the intervention was a means to refer systems and structural issues to higher levels, and, by virtue of new strategies alliances and relationships, better positioned for systems response.

While encouraging, initial impacts should be considered relative to operational contexts and challenges. Low levels of CHW integration remain in the formal PHC system. WBPHCOT implementation has been slow and uneven, and there is low coverage [16, 52]. There is a lack of national leadership and financial support, poor governance, low employment status and pay, political interference, inadequate supervision and support, particularly in terms of links to facilities, and roles are poorly defined [16, 52]*.* As of 2020, the provincial DoH has established less than half of the planned WBPHCOTs (235 out of 560 [42%]). While more progress is needed, new policy commitments are driving integration. The provincial strategic plan 2020–24, aims to absorb all 6119 CHWs currently funded by CBOs into government contracts [53].

Overall, professional labour shortages for CHWs and PHC are extensive [54]. In 2021, the DoH reported recruiting an additional 985 CHWs across the country, bringing the total to 48,443 [55]. The following year, however, there was no increase in recruitment owing to COVID and budget constraints [56]. Moreover, the health promotion and prevention focus in PHC Re-engineering overlooks the need for CHW-led curative care, e.g., for malaria and childhood pneumonia, diarrhoea and acute malnutrition [57]. It has been estimated that South Africa would need closer to 400,000 CHWs to redress this balance [57]. South Africa’s disease profile is one of multiple, transitioning burdens of socially patterned illness and disease [40]. Expanded CHW training needs to be *integrative*: of chronic health conditions as well as their social determinants [58].

Fundamentally, the evaluation underscored that CHWs need to integrate with *both* communities and services. This necessitates skills and competencies to navigate varied relationships in a sustained, strategic manner [59]. Acknowledging a limited evidence-base, WHO guidance sets out the need for core CHW competencies in communication, and community engagement and mobilisation [60]. Recommended training modalities include: theoretical and practical, with priority on practical experience; face-to-face and e-learning, with priority on face-to-face; training in or near the community; in appropriate languages; positive training environments; and inter-professional approaches [60]. There is recognition of the need for evidence on certification or contracting and career progression, as well as contextualised, realistic HPSR understandings of what works, how, with whom, to what extent and within specific health systems contexts and circumstances [60, 61].

**Fig. 3:**
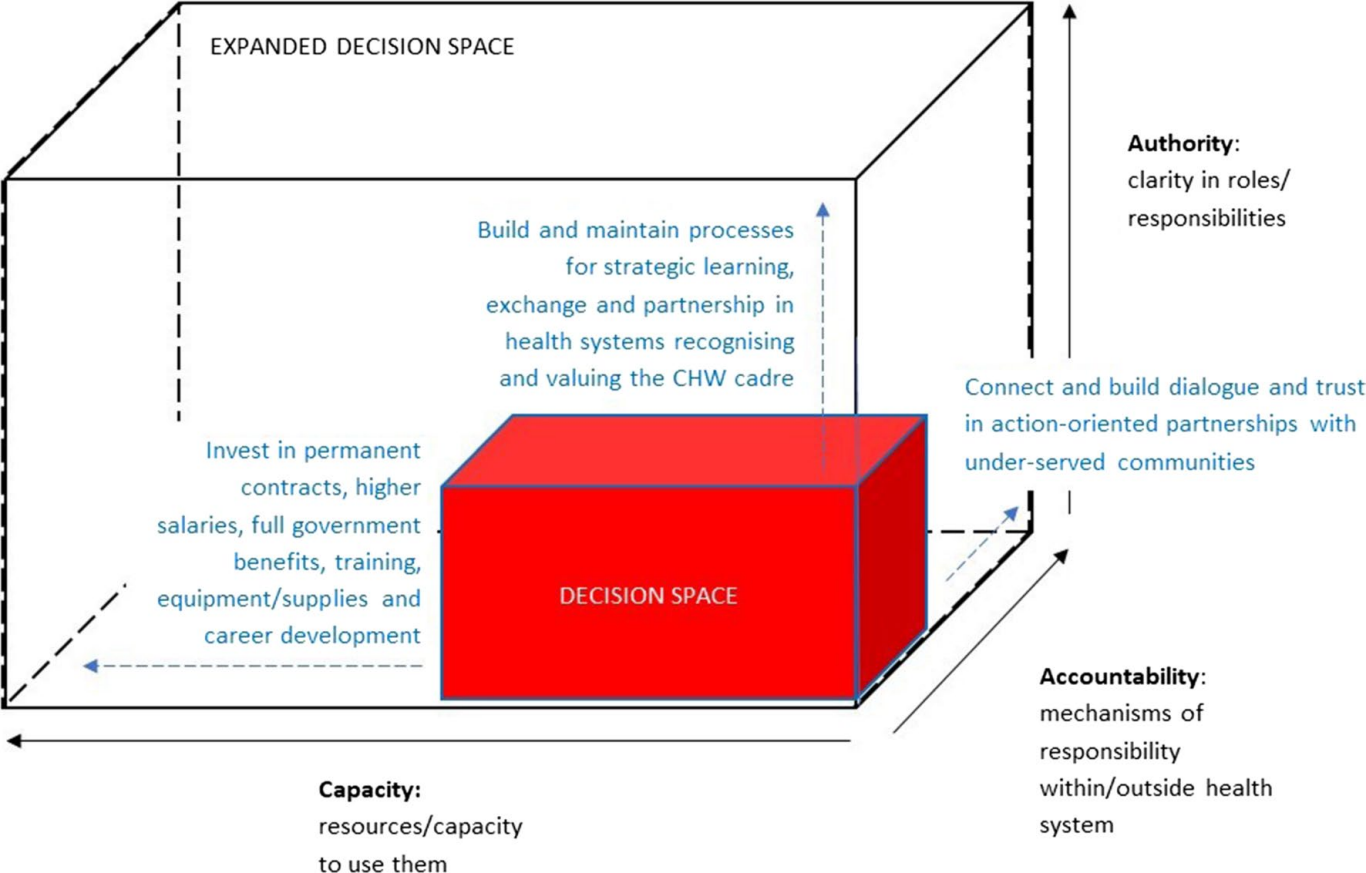
Dimensions of increasing decision space

The study contributes to these gaps, as well as gaps identified by Roman et al. on understanding and supporting the extent of power for local decision-making as part of decentralising health reforms [33]. A limited number of studies explore interactions between accountability mechanisms, resources and organisational capacity and how this influences available decision space. Understanding these functions and how the components interact has the potential to improve the feasibility of functional organisations for CHWs, and improve policy implementation. Synergy across the components was vital in enabling or constraining CHW decision space. In this analysis, accountability mechanisms were present but need to be coupled with improved organisational recognition and, critically, improved resources in a range of areas (Fig. 3). Financial, human, administrative, technical, and organisational resources are required to ensure success, scale-up and sustainability and, ultimately, improved local decision-making in the health sector [62, 63].

Decentralisation is a response to disinvestment, human resources crises and structural disadvantage in public institutions in LMICs. The depth and extent of which exist across the health system; in how people and their professional roles are subjectively perceived and organisationally valued, and how they are materially resourced. In theory, decentralisation has the potential to support the South African health system to deal with protracted epidemiological transition, entrenched health inequalities, and an evolving situation in relation to PHC re-engineering, and COVID-19 [64]. In practice, HPSR approaches expanding everyday leadership and resilience [65], such as and including widening CHW decision space, have the potential to realise policy goals of close-to-people care. The analysis suggests that mutually supportive, bottom-up approaches can support PHC reforms by consolidating and harmonising strengths at different levels of the system. Structural challenges should not be overlooked. As Roman et al. point out, there will be little change, where decision space is unavailable [33]. There is an immediate and fundamental need to shift CHW temporary contracts to permanent government positions with higher salaries, pensions, leave and sick pay, to improve working conditions and resourcing, and for whole systems improvements in the organisational valuing of the cadre [29, 30].

### Methodological reflections

Decision space is an approach to understand how decentralised health systems operate. The framework has not been applied in great depth to CHW roles and functions. The framework structured the evaluation and supported increased understanding of health workers in lower levels of the system, their difficulties and bureaucratic challenges faced. Our approach incorporated different lenses on community health systems encompassing health systems ‘building blocks’, social relations in complex systems, and front-line standpoints [66]. Future work should expand critical analyses on decentralisation and addressing the organisational valuing of the cadre.

Otherwise, the analysis was based on a small sample drawn from the surveillance area, which is reasonably representative of the district and province. This study was concerned solely with CHW perspectives. This was owing to their marginalisation in the system, their being the primary recipients of the training, and in response to calls from the WHO for more research on different training styles, CHWs as active change agents, and elevating CHW voices [5]. Acknowledging the training as inherently mutual, the analysis complements other studies, evaluations and learning from the perspectives of community and health systems stakeholders [38, 43, 44]. Extending and integrating evaluative evidence base on participatory learning interventions is a priority in future.

In terms of the process, stability, predictability, and trustworthiness were important features of the platform during the global public health emergency in 2020/21. The HDSS institutional base conferred legitimacy on the team and platform owing to its established presence and trusted relationships with rural communities and the district health system over 30 years [41]. The research team University and DoH staff, with data collection and analysis led by South African and Ghanian clinical researchers in Universities. As CHW’s experiences were documented, we empathised with female colleagues, in their bid to provide for their families and communities, and in terms of the expectations from resilience and commitment in the face of multiple systems constraints and new threats from a global pandemic.

Codesign was critical. Strategic partnerships between CHWs and health system are important to overcome weakness in CHW programmes [67]. The process was codesigned to be of practical relevance to health systems actors and every design decision was made to provide and expand utility to end-users needs and preferences to support uptake. Formalising, sustaining and embedding the process take time and space with multi-sectoral colleagues adapting methods for use in districts. Continued cooperative working is key as the model is adapted for inclusion into routine health systems functions.

Most CHWs have now returned to 'usual' work patterns, with some facilities retaining a 50:50 split between the clinic and community (personal communication, 31 January 2023). ‘Post-COVID’, further challenges play out related to widening and deepening of inequalities [68], human resources for health crises [69], and multiple, intersecting burdens of disease and chronic illness [40, 70]. These affirm the need for new forms of real-time health systems and policy learning, that are inclusive and embrace diverse forms of evidence and learning, combining insights from implementation experience with policy and planning [71]. Our study demonstrates that building meaningful partnerships between CHWs, communities and policy-makers is possible and has the potential to confront and transform the underlying structures of health inequalities.

## Conclusion

CHWs face entrenched systems challenges in terms of recognition as a permanent, central feature of PHC. During the early part of the COVID-19 pandemic, CHWs in this setting experienced shifting roles and remits, chronic professional labour shortages, and limited training, supervision and mentorship. The training intervention focussed on building community mobilisation competencies through rapid PAR. The training built new skills and competencies, new strategic community and facility-based alliances, and supported respect for and recognition of CHW roles, value, and contribution at higher levels of the system. The process was subsequently scaled across the sub-district. The study adds to the literature on decision space and highlights potential areas for future research, particularly in provision of resources, organisational capacity and strengthening accountability, and for critical analysis of decentralisation and its role in health systems improvements.

### Supplementary Information

**Additional file 1.** VAPAR Mpumalanga health policy and systems research learning platform, Mpumalanga department of health. Community health workers community mobilisation training manual (english version). VAPAR Mpumalanga health policy and systems learning platform; 2022. https://www.vapar.org/_files/ugd/532615_d50539e3dc82416980f5c4732e11eb10.pdf.

**Additional file 2.** VAPAR Mpumalanga health policy and systems research learning platform, Mpumalanga Department of Health. Community health workers community mobilisation training manual (siTsonga version). VAPAR Mpumalanga Health Policy and Systems Learning Platform; 2022. https://www.vapar.org/_files/ugd/532615_1384f348491e422c9080e4d28e70d4a9.pdf.

## Data Availability

Data are available from the research team upon reasonable request. A non-author point-of-contact to field future requests (where authors are not available) is achds@abdn.ac.uk. Public deposition of the data set would be in breach of the data management plan (DMP) within the study protocol approved by the research ethics boards in South Africa, the UK, and the permission for the study granted by the provincial health research committee, as well as the DMP within the funding proposal and the conditions upon which the research funding was granted.

## Abbreviations

CBO: Community-based organisation
CHW: Community Health Workers
COVID-19: Coronavirus Infectious Disease 2019
DCST: District Clinical Specialist Team
DHS: District health system
DOTS: Directed observed treatment (TB)
GBP: Great British Pound
HBC: Home-Based Care
HDSS: Health and Socio-Demographic Surveillance System
HIV/AIDS: Human Immuno-Deficiency Syndrome/Acquired Immune Deficiency Syndrome
LMIC: Low- and middle-income countries
OM/OPM: Operational Managers
OTL: Outreach Team Leader
PAR: Participatory action research
PHC: Primary health care
PPE: Personal Protective Equipment
UHC: Universal health coverage
VA: Verbal autopsy
VAPAR: Verbal Autopsy with Participatory Action Research
WBPHCOTs: Ward-based Primary Healthcare Outreach Teams
ZAR: South African Rand
