# Correction: Expanding Community Health Worker decision space: learning from a Participatory Action Research training intervention in a rural South African district

**DOI:** 10.1186/s12960-023-00863-z

**Published:** 2023-10-05

**Authors:** Lucia D’Ambruoso, Nana Akua Abruquah, Denny Mabetha, Maria van der Merwe, Gerhard Goosen, Jerry Sigudla, Sophie Witter, the Verbal Autopsy with Participatory Action Research (VAPAR)/Wits/Mpumalanga Department of Health Learning Platform

**Affiliations:** 1 Aberdeen Centre for Health Data Science, School of Medicine, Medical Sciences and Nutrition, and Centre for Global Development, School of Education, University of Aberdeen, Aberdeen, Scotland, UK; 2 MRC/Wits Rural Public Health and Health Transitions Research Unit (Agincourt), School of Public Health, University of the Witwatersrand, Johannesburg, South Africa; 3 Department of Epidemiology and Global Health, Umeå University, Umeå, Sweden; 4 Public Health, National Health Service (NHS) Grampian, Aberdeen, Scotland, UK; 5 Department of Global Health, Stellenbosch University, Stellenbosch, South Africa; 6 The University Hospital, Kwame Nkrumah University of Science and Technology, Kumasi, Ghana; 7 Cochrane South Africa, South African Medical Research Council (MRC), Cape Town, South Africa; 8 Maria Van Der Merwe Consulting, White River, South Africa; 9 Mpumalanga Department of Health, Mbombela, South Africa; 10 Institute for Global Health and Development, Queen Margaret University, Musselburgh, Scotland, UK

**Correction: Human Resources for Health (2023) 21:66** 10.1186/s12960-023-00853-1

Following publication of the original article [1], an error was identified in the Acknowledgements

section. The updated acknowledgements is given below and the change has been highlighted in **bold typeface**.

**Acknowledgements**

The authors dedicate this paper to Professor Peter Byass, who died suddenly in 2020. Peter’s death had profound personal and professional impacts on the team. His death has also galvanised us to continue his work on information systems in South Africa and globally. The authors would also like to thank community, government, and non-governmental stakeholder participants for agreeing to be part of the process, and for sharing their time, knowledge, and perspectives. Thanks also to the Verbal Autopsy with Participatory Action Research (VAPAR) team and staff of the Medical Research Council (MRC)/ Wits Rural Public Health and Health Transitions Research Unit (Agincourt), especially Mr Simon Khoza, Dr Rhian Twine and Professors Stephen Tollman and Kathleen Kahn. Thanks to **Hanna Kruger** for preparing the Google Map contained in Fig. 1.

The original article [1] has been corrected.
