# Supplementary material for: Expanding Community Health Worker decision space: learning from a Participatory Action Research training intervention in a rural South African district
Source: Hum Resour Health. 2023 Aug 18;21:66. doi: 10.1186/s12960-023-00853-1 (PMC10439531; doi:10.1186/s12960-023-00853-1)
Supplement: Supplementary file 2 — Additional file 2. VAPAR Mpumalanga health policy and systems research learning platform, Mpumalanga Department of Health. Community health workers community mobilisation training manual (siTsonga version). VAPAR Mpumalanga Health Policy and Systems Learning Platform; 2022. https://www.vapar.org/_files/ugd/532615_1384f348491e422c9080e4d28e70d4a9.pdf. [file 12960_2023_853_MOESM2_ESM.pdf]

Mpumalanga Health Policy and Systems Learning Platform

Community Health Workers Community Mobilisation TRAINING MANUAL

Verbal Autopsy with Participatory Action Research (VAPAR)

vapar

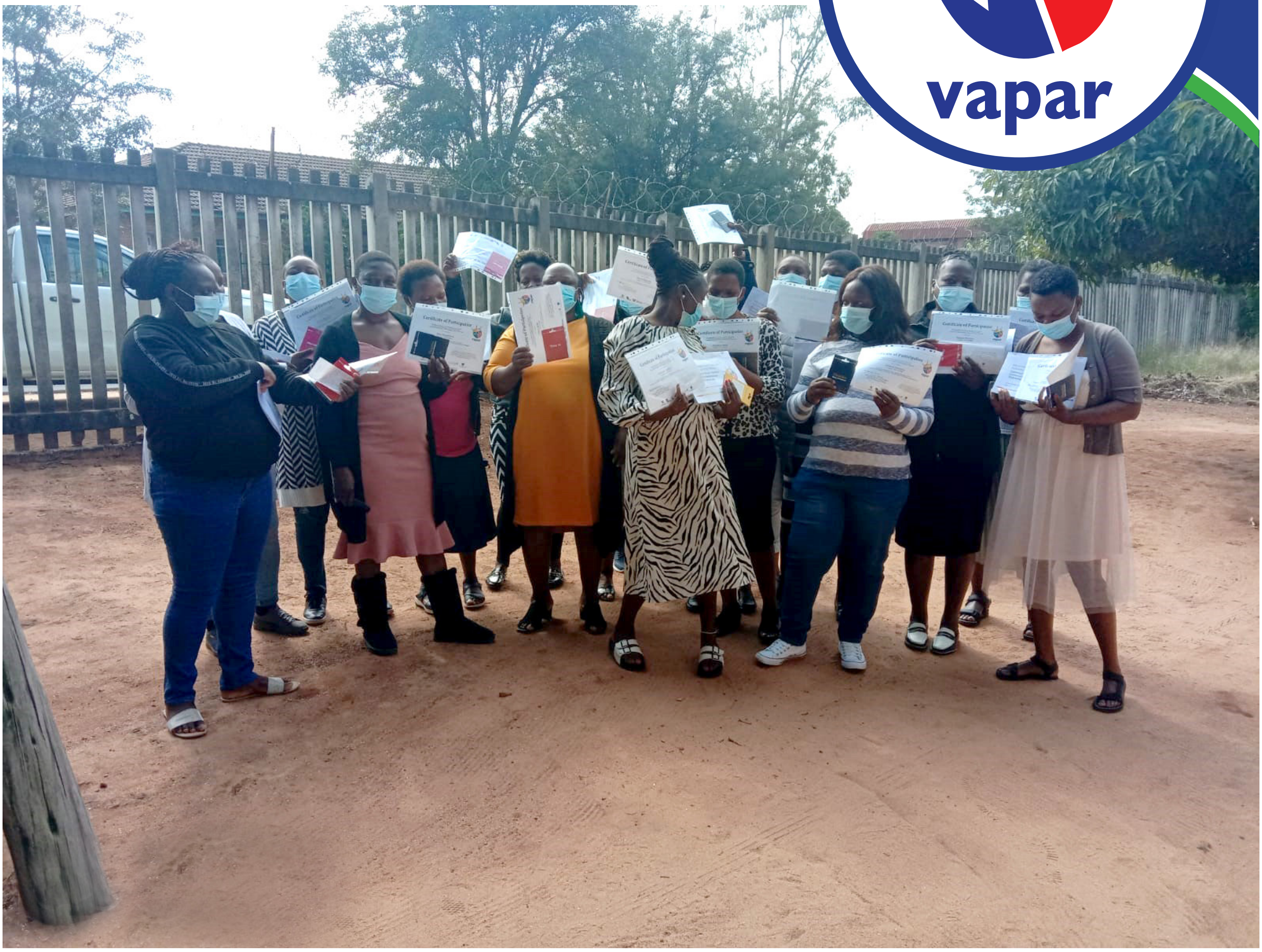

November 2022

health
MPUMALANGA PROVINCE
REPUBLIC OF SOUTH AFRICA

Mpumalanga Health Policy and Systems Learning Platform

Community Health WorkersCommunity Mobilisation

TRAINING MANUAL

The Mpumalanga Health Policy and Systems Learning Platform addresses exclusion from access to health systems by connecting service users and providers to generate and act on research evidence of practical, local relevance.

The platform has been developed through the research programme VAPAR (Verbal Autopsy with Participatory Action Research) progressed by the MRC/ Wits Agincourt Rural Health and Health Transitions Research Unit.

MRC Wits/Agincourt hosts the Health and Socio-demographic Surveillance System (HDSS) within which we develop health statistics, using verbal autopsy, and work with local communities to expand local knowledge on health priorities.

We gratefully acknowledge members of the independent project steering committee of the VAPAR programme, who peer reviewed the April 2022 version of this training package.

(cc) VAPAR 2022

Licensed under the Creative Commons LicenseAttribution 2.5

http://creativecommons.org/licenses/by-nc-nd/2.5

Please feel free to copy or distribute to anyone you think would benefit. If you're interested in more information on community participation and rapid participatory action research methods, visit our website at **www.vapar.org** and let us know your thoughts.

FOREWORD

"The manual, from the department's perspective, particularly at the sub-district level, inspires a great sense of pride about the realisation of the possibility of building capacity for this cadre of emerging health care workers in SA. The manual will go a long in providing a practical and a formal tool to guide Community Health Workers through their day to day work with communities."

Mr R Mabika

Acting sub-district PHC manager: Bushbuckridge

"Nkatsakanyo lowu wa matirhelo (Manual), wu tisa kuti nyungubyisa loku kulu swinene eka Ndzawulo ya Rihanyu, ngopfu-ngopfu eka xifundzha-ntsong xa Bushbuckridge, hikuva wu tisa ku humelela hitlhelo ro antswisa vutivi byo tirha eka ntlawa lowu wa vatirhi va swarihanyu va Ma-Community Health Workers eAfrika-Dzonga. Nkatsakanyo lowu wa matirhelo wuta pfuna swinene ku letela Ma- CHW's hita matirhelo ya siku na siku eka miganga leyi vatirhaka na yona."

Mr R Mabika

Acting sub-district PHC manager: Bushbuckridge

INTRODUCTION

WHAT IS THIS MANUAL?

Manual lowu wu katsa ma thulusi lawa ya nga tirhisiwaka hi ma Community Health Workers (CHWs) la wa ya tirhaka tani hi Ward Based Primary Healthcare Outreach Teams (WBPHCBOTs).

Manual lowu wu kongome ku pfuneta ma CHWs ku hlanganisa ntlawa wa ti community stakeholder, ku yisa ehenhla kumbe ku hlamula eka swilo leswi faneleke ku rhangisiwaka emhahlweni loko swita eka swiphiqo swarihanyo, ku twisisa ntumbuluko wa swivilelo na leswi swi taka hileka ti stakeholders taleka community, na ku sungula mbhurisano xikan'we naku landzelerisa swilo leswi swi nga endliwaka eka ti communities na leka health system nati public services leti ti n'wana.

Ma CHW hi vona vo sungula lava va kotaka ku hlanganaka na ti communities xikan'we na vale ka health system, na swona hi vona va faneleke ku fambisa mi mbhurisano eka community, hi ku seketela ti structures leti ti nga kona leti ti fambisaka mi mbhurisano eka community, ku fana na ti district health councils and clinic and community health centre committees, ku katsa na vayimeri va leka ti communities.

Hi tekela nhlokweni ma term yo fana na: community participation, community engagement and community mobilisation. Hi xikongomelo xa manual, rito 'community mobilisation'I term leri ri tirhisiwaka eka ti local health system leri ri hlamuselaka maendlelo yaku rhamba ti community stakeholders, ku yisa ehenhla/ ku tlakusa na ku twisisa xi kan'we na ku hlamula eka swiphiqo swarihanyu leswi va vilelaka ngopfu hi swona, ku endla mbhurisano na ku teka xiendlo ku tlhela ku landzelerisiwa eka community na leka health system.

WHO WILL BENEFIT?

Manual lowu wu endleriwe ku seketela ma CHWs ku antswisa rapid research methods naku fambisa ti community mobilisation activities. Ma endlelo lawa ya tlhele ya endleriwa ku vuyeriwa ka vanhu eka communities.ti thulusi leti ti endleriwe ku seketela ti communities ku endla, ku twisisa na ku lulamisa swiphiqo leswi swi nga kona swa rihanyo hi ku angarhela naku seketela ndlela leyi mfumo wu endlaka swi boho hi yona loko swita eka rihanyo eti ndhawini ta local.

HOW DOES IT WORK?

Manual lowu wu katsa ti thulusi ta 5 leti ti tirhisiwaka eka participatory action research (PAR) ku komba na ku twisisa xi kan'we na ku lulamisa swi phiqo leswi swi nga kona swa rihanyu eka ti rural communities.

Ti thulusi leti ti tirha hi ndlela yo landzelelana: (1) ku komba xiphiqo lexi xi nga kona; (2) ku lava vu xokoxoko mayelana na xiphiqo lexi; (3) ku twisisa swivangelo na swita ndzhaku swa xiphiqo lexi; (4) ku endla map laha ku fakiwaka vanhu lava va nghenelelaka eka xiphiqo lexi na switandzhaku leswi swi nga kona eka xiphiqo lexi; (5) ku pulana xiendlwati na ti stakeholders taku hambana hambana.

The tools are underpinned by a four main principles (described in more detail on page 8):

1. **'homogenous group'** – mintlawa leyi yi hlanganisaka hi swiyimo na swivilelo swin'we;
2. **'subjective perspectives'** – ku twisisa ntokoto wa vanhu;
3. **'collective validation'** – ku tekeriwa nhlokweni ntsena lexi xi hlawuriweke hi ntlawa;
4. **'no delegation'** – lava va tirhaka hi mhaka leyi ku vulavuriwaka hi yona hi vona va nga ta rhangela.

The content and messages are aligned with the National Department of Health policies and training curriculum for CHWs.

WHAT ARE WE AIMING FOR?

Maendlelo lawa ya endleriwe ku tlakusa ti local action eka social determinants of health, ku seketela ku tumbuluka ka vuxaka bya ku twisisana na ku tshembhana exikarhi ka ti communities na ma CHWs, na ku endla leswaku ku va na ku tekeriwa enhlokweni ka role ya ma CHWs na WBPHCOTs in local health governance structures such as clinic committees and district health management teams.

Adapted from: Cavestro, 2003; and Loewenson et al, 2006

CONTENTS

GENERAL

Definitions

Principles

Recruiting participants

Convening stakeholders

Ground rules

Facilitation

IDENTIFYING HEALTH CONCERNS

Tool 1 / Workshop 1: RANKING AND SCORING

Workshop leyi na thuli leyi swita kotisa ma CHWs na ti community stakeholders ku komba ti nhlokomhaka ta swa rihanyo leti ti fambelanaka na community.

Ti nhlokomhaka to tala ta vuriwa kutani ku hlawuriwa yi n'we leyi ku nga yona yi tekeriwaka e nhlokweni eka local level hi ku tirhisa thuli leyi vuriwaka Scoring.

Maendlelo ya scoring ya endliwa hambi kuri ka mbirhi ku ri karhi kuri na ku vulavurisana ku tlhela kuva na ku pfumelelana emakumu.

UNDERSTANDING THE ISSUES

Tool 2 / Workshop 2: PROBLEM TREE

Workshop na thuli leyi swita kotisa ma CHWs na ti community stakeholders ku twisisa na ku ntlhantlha nhloko mhaka leyi yi nga hlawuriwa hi ndlela yo tirhisa ku vote.

Hi ku facilitate na ku vulavurisana hi ku tirhisa xifaniso xa nsinya lexi xi nga ta voniwa hi u n'wana na u n'wana, va ngheneleri va ta komba swi vangelo na switandzhaku leswi swi nga kona kuya hi vuxaka eka ti level to hambana, laha ku ngata tekeriwa nhlokweni mavonelo yo hambana hambana lawa ya nga ta nyikiwa.

MAPPING ACTORS AND IMPACTS

Tool 3 / Workshop 3: VENN DIAGRAM

Workshop leyi na thuli leyi swi ta kotisa ma CHWs na ti community stakeholders u twisisa switandzhaku na vanhu lava faneleke ku nghenelela loko swita eka ku lulamisa xiphiqo xo karhi.

Ku endliwa ka vuxaka exikarhi ka vangheneleri vo hambana hamba swita tekeriwa nhlokweni kumbe ku endliwa.

Leswi swita pfuna ekaku ku komba ti internal na ti external organisations leti fambelanaka na xiphiqo lexi xi hlawuriweke na leswaku swi fambelana njani loko swita eka nhlohlotelo (influence) na ku tirhisana xi kan'we na ku hlangana.

PATHWAYS TOWARDS CHANGE

Tool 4 / Workshop 4: ACTION PATHWAY

Workshop leyi na thuli leyi swi ta kotisa ma CHWs na ti community stakeholders ku komba ti ndlela hi nkwato leti valava kuti teka loko swita ekaku lulamisa xiphiqo lexi na ku endla hi ndlela yo landzelelana leswaku swilo leswi va nga swi fikelela njani va tlhela va komba na vanhu lava va nga ta rhangela ti action (swiendlwa) leti.

Thuli ya action pathwayyi endleriwe ku komba ndlela leyi mi ngayi landzelelaka ku fikelela swilo swo karhi.

Ku pulana, ku endla na ku landzelerisa swa endliwa.

COLLECTING AND ANALYSING VISUAL DATA

Tool 5: PHOTOVOICE (for use in any workshop)

Thuli leyi yita kotisa ma CHWs na ti community stakeholders ku endla vumbhoni bya ntokoto wa vona hi ku teka swinepe, va tlhela va yi thisisa ku seketela leswi va swi kumeke lo va tirhisa thuli ya ku sungula ku fika eka yavu mune

Vangheneleri va ta dyodzisiwa ka tsongo hi ku tirhisa ti camera ku teka swinepe, va ta dyondzisiwa hi papila ra mpfumelelano, ku tsala nhloko mhaka ya xiphiqo lexi xi nga leku lulamisiweni, u tlhela u tsala xihungwana mayelana na leswi swi nga le ka xinepe swi fambelana ni leswi u nga vona swona.

Swinepe swa kombisiwa swi tlhela swi vulavurisiwa eka meeting, ku tlhela ku endliwa marito ya leswaku xinepe xi ngava xi hlamusela yini hi ku fambelana na nhlokomhaka leyi yi hlawuriweke.

NEXT STEPS 'GIVING VOICE TEETH'

Uya emahlweni na ku hlanganyela na health system/other services:

Ku endla vuxaka byaku tshembhana eka ti communities

Ku ti hlanganisa na ti district health systems, primary health care (PHC) planning and reviewku ti hlanganisa na ti stakeholders ta ku hambana hambana ku lulamisa swiphiqo leswi swi fambelanaka na social determinants

GENERAL: DEFINITIONS

COMMUNITY: ntlawa wa vanhu lava va tshamaka eka ndhawu yi n'we kumbe lava va ngana mahanyelo yo yelana, community ya ncinca hi mikarhi yo tala e.g. HIV community, LGBTQI community.

COMMUNITY MOBILISAITON: swi voniwa tani hi maendlelo laha ntlawa kumbe mintlawa ya vanhu yi pfuniwaka ku humelerisa swi laveko swa vona na ku tlhela va tirhisana ku teka goza eka swi laveko sweleswo. Swi jeketela ku nghenelela ka vanhu hi voxe leswaku vata kota ku fikelela magoza lawa vaya tekeke na ku tlhela va hlanganyela

COMMUNITY HEALTH WORKER: leswi swi vula munhu u n'wana na u n'wana loyi a tirhaka hi swa rihanyo, lo yi a nga hlawuriwa a tlhela a trainiwa ku tirhela eka community. Hi vona va hlanganisaka ti community na ti various health and social development services. Va seketela vanhu valeka community kuva va nga teka swi pimelo ma yelana na rihanyu ra vona, va tlhela va seketela miminti leyi yi nga siwaneka hikuva va va vhakela e makaya.

STAKEHOLDER: Marito yo fana na 'participants 'na 'stakeholders' ma tirhisiwa ngopfu laha ka manual lowu. Leswi swi vulaka vanhu lava vangatava va ngenelela eka maendlelo lawa kumbe lava va ngata katsiwa eka maendlelo lawa

COMMUNITY PARTICIPATION IN HEALTH: va ngheneleri va fanele ku vulavula kumbe ku katsiwa eka xiboho xi n'wana na xin'wana lexi xi kongomeke vona. Laha swi nga ta laveka leswaku va teka magoza eka swi n'wana va tlhela va landzelerisa magoza lawa vaya tekeke.

EMPOWERMENT: ku nyika vanhu matimba yo kota ku hluvukisa xa n'wa nchumu hi ndlela yo hlanganyela.

SOCIAL DETERMINANTS OF HEALTH: hi swi yimo leswi vanhu va beburiwaka eka swona, leswi va kulelaka eka swona, va tirhelaka eka swona, va hanyaka eka swona va tlhela va kulela eka swona,swi swi hlohlotelaka xiyimo xa rihanyu. Xikombiso swi katsa: income and social protection (grants); education; housing, basic amenities; the environment; unemployment and job insecurity; and food insecurity.

WHAT IS COMMUNITY MOBILISATION?

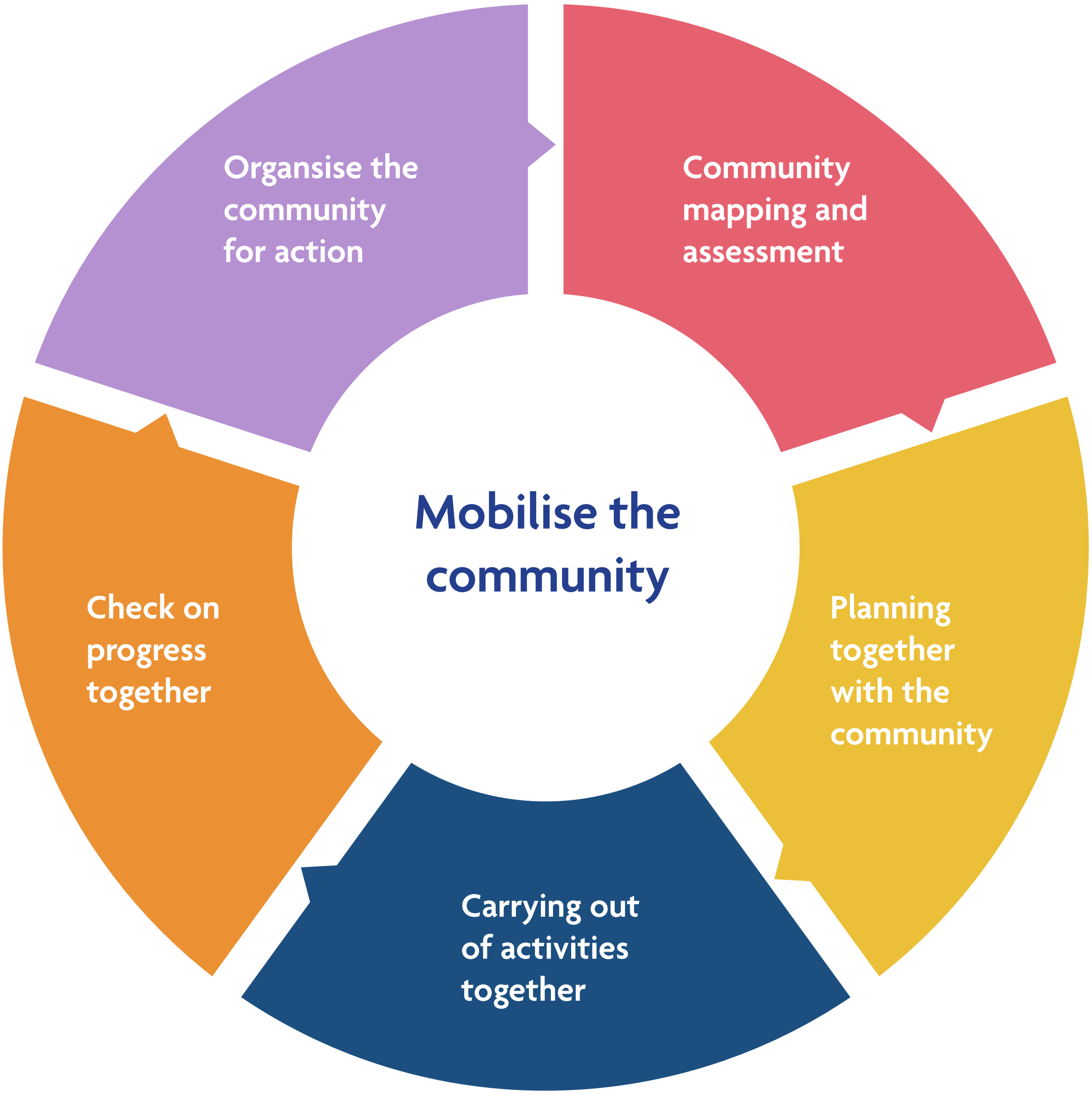

health
Department:
Health
REPUBLIC OF SOUTH AFRICA

I YINI COMMUNITY MOBILIZATION?

Mobilize the community-Hluvukisa tiko.

Organize the community for action-Lulamisa tiko ku endla goza

Check on progress together-Langutisisani xikan'we leswi swi endliweke.

Carrying out of activities together-Ku endla migingiriko xikan'we.

Planning together with the community-Ku pulana xikan'we na tiko.

PRINCIPLES

HOMOGENOUS GROUP

Group with shared conditions, interests, and concerns

Ma thulusi lawa ya nga la ya fanele yatirhisiwa hi mintlawa leyi yi nga homogeneous. Loko hi vula leswi hi vula mintlawa leyi yi ngana swiyimo leswi swi fanaka, ku tsakela loku ku fanaka na ku vilela kun'we, na swona kuva mintlawa ya vanhu lava rito ra vona ri nga twiwiki loko swita eka maendlelo yo lulamisa swilo naloko swita eka timhaka ta service delivery. Mintlawa leyi yi katsaka ma CHWs na swona va fanele kuti yimisela ku yimela mavonelo yo karhi eka xiphiqo xo karhi, xikombiso: Vantshwa, vana va leka adolescents na vanhu lava va ngana ntokoto loko swita eka swilo swo fana na ti service delivery. Leswaku vata vulavula hi swilo leswi fambelanaka na byala na ku xanisa swidzidziharisi, na mintlawa yi n'wana leyi yi hlanganeke leyi yi katsaka (traditional leaders, family members, women of reproductive age) yi nga endliwa ku burisana hi xiphiqo lexi xi nga kona xo fana na xaku pfumaleka na mati yo basa eka community.

NO DELEGATION

Those dealing with the issue are the primary actors generating information

Community mobilisation I ku ngenelela ka vanhu lava va twaka ku vava ngopfu hi xiphiqo xo karhi. Hikokwalaho lava va lulamisaka xiphiqo xelexo hi vona va ngheneleri lava kulu lava va faneleke ku humesa vuxokoxoko lebyi fambelanaka na xiphiqo xelexo, va tlhela va burisana na ku teka goza ekaku lulamisa xiphiqo lexi.

SUBJECTIVE PERSPECTIVES

People's individual opinions and experiences are central

mavonelo ya munhu u n'wana na u n'wana na ntokoto wa vona I swa nkoka. Hiku tirhisa ma thulu lawa ya nga ka manual lowu, mavonelo ya vanhu ya tsariwa kutani ku burisaniwa hi wona, ku tlhela ku tekiwa mavonelo ya community na magoza lawa ya nga endliwaka ku lulamisa xiphiqo.

COLLECTIVE VALIDATION

Only those issues that are recognised by group are registered

Maendlelo lawa ya tisa mavonelo ya vanhu ya hlanganisa na mintokoto leyi vanhu va ngana yona, hi ndlela yo vulavurisana kahle hiku xiximana. Kuna principle leyi yi nga vekiwa ya leswaku ku tekeriwa nhlokweni ntsena leswi vulavuriwaka hi ntlawa.

Adapted from: Loewenson et al, 2014

GROUND RULES

Facilitator hi yena angata veka milawu ku pfuna ekaku endla leswaku mi mbhurisano leyi yi endliwa hiku xiximana na ku endla leswaku kuva na ku twisisana na ku tshembana eka vanhu hinkwavo lava va nga ngenelelaka.

PEOPLE MATTER

Vanhu hi vona masungulo ya maendlelo lawa/nongonoko lowu. Ku endla leswaku vanhu va titwa va tekeriwa e nhlokweni. Ku tekela nhlokweni ti ndhawu leti ku ngata khomiwa ka tona mi nhlangano. Le swaku ti ngava ti tshamisekile leswaku va ngheneleli va va eka tona? Ti ngava tiri ti ndhawu leti nga kotata ku nyinka space leswaku kutava na mimbhurisano yo tika? xikombiso, ku susa swi tulu kumbe switirhisiwa leswi swi onhakeke.

NON- THREATENING

Ku xixima mavonelo ya vanhu na mintirho ya vona eka community. Ku pfumelela mavonelo yo hambana hambana, ndhavuko na leswi vanhu va nga hi xiswona, ku hlohlotela ku twisisa leswaku ku fanele ku xiximiwa ma vonelo yo hambanahambana na leswaku ku na matimba ekaku endla xilo hintlawa.

NO BLAME

Ku endliwa leswaku swi vilelo swa vanhu swa twiwa. Ku amukela leswaku kuna vuxaka byaku tika eka ti communities na le xikarhi ka ti communities na varhangeri va kona, kambe hiku famba ka nkarhi, ku lehisa mbilu na ku vulavula hiku xiximana ku ngava na ku fikelela ka ku navela kun'we na vuxaka bya ku tshembana byi nga kula.

RELAXED ENVIRONMENT

Ku hlohlotela vanhu leswaku va fanele ku vulavula hi mintokota ya vona na ku tlhela va lava swi ntshuxo.

RESPECT

I swa nkoka leswaku ku fanele ku va na nkarhi na ndhawu ya ku yingisela hi ku xixima va n'wana ku ngari na kuva kavanyeta. U n'wana na u n'wana u ta nyikiwa nkarhi wa ku vulavula a nyika mavonelo ya yena. Va ngheneleri va nga komberiwa, xikombiso, ku yimisa mavoko leswaku facilitator a kota ku endla leswaku u n'wana na u n'wana u kuma nkarhi wo vula vula a nyika mavonelo ya yena.

INFORMATION

Ku tirha hi ndlela leyi yi xiximaka ntokoto wa vangeneleri. Ku tisa mihlovo hlovo ya data, leyi yi katsaka data ya tinomboro na data ya swinepe.

DEMANDS ON FACILITATORS

Ti facilitators ti fanerile ku endla leswaku vanhu hinkwavo va vulavula, leswi swi kota ku aka vuxaka exikarhi ka vanhu hinkwavo.

RECRUITING PARTICIPANTS

RECRUITMENT PROCEDURE

Ma CHWs ya nga recruit ti participants ya tirhisa tindlela to hambana hambana to fana na door-to-door outreach, or community presentation and activities. Hi nkarhi waku recruit ma CHWs ya fanele ku nyika nkomiso wa maendlelo lawa, va highlight nkongomelo wa maendlelo lawa. I swa nkoka ku kombela va ngheneleri leswaku va nyika tinomboro ta vona ta riqingho na vuxokoxoko mayelana na ndhawu leyi va tshamaka eka yona leswaku vata kota ku va landzelerisa na ku vulavula na vona kuya emahlweni.

Tani hi xiphemu xa training leyi, ma CHWs yata recruit ti participants ku suka eka ti communities, lava vanga tshama va nghenelela eka migingiriko leyi yi endlekaka eka community, lava va tshamaka vari kona emakaya na swona vana ku tsakela ka swilo leswi swi fambisanaka na nhlokomhaka ya swa rihanyu leyi yi hlawuliweke eka workshop yo sungula.

Vanhu lava va tshamelaka kuka va nga koti ku kufikelela ti health services, kumbe lava va sukaka ka swiyimo swa vusiwana, va fanele ku rhangisiwa ku nghenelerisiwa. Loko se mi va kumile vangheneleri, va byeleni hi nkarhi ni ndhawu ya workshop, mi tlhela mi va kambela leswaku va ngava va yi kumile ndhawu yo khomela ka yona workshop. Ku vulavula hiti mhaka ta mali ya transport.

Vangeneleri va fanele ku nyikiwa papila leri ri tsariweke vuxokoxoko bya maendlelo lawa, laha ku nga tsariwa nkongomelo wa maendlelo lawa, ku tsariwa na switandzhaku na ku vuyeriwa loku ku nga kona kakuva va ngenelerile. Swi kahle na kambe ku nyika vangeneleri nkarhi wo ringana 72 hours kuva va hlaya papilla leri va tlhela vari twisisa na ku vutisa swi vutiso va ngasiza va pfumela ku ngenelela.

EXAMPLE INVITATION LETTER

Xikombiso xa papilla ra xirhambo.

__Introduction__: Hello, my name is____. I'm from the [name] clinic.

__Background__: We worked in the area in to learn about what people know about different health conditions and what kind of health services can help to respond to the needs of this community. We want to continue and extend these partnerships to support this community to become more involved in activities and decisions that affect health and health services here. To do this, we are forming a Village-Based Discussion group in this village. The group will be made up of community members (e.g., village health workers, traditional healers, women of reproductive age, family members, village leaders etc.). We would like to invite you to be a member of the group for this village.

__Involvement__: Group members will be invited to a series of meetings. The meetings will each take about 1.5 hours and will be held in this village. At the meetings we will talk about health conditions that are relevant here. We will talk about why people died and how, what it is like to have specific health problems in this community. We will also identify what kind of health services could help with the health needs of people here. This will be useful information for community members and help the Department of Health to improve community and health services, and for village based discussion groups to reflect on and learn from the process relative to any actions taken on the basis of it by the health authority or other groups. We will also provide digital cameras to photograph local areas to inform the discussions and illustrate the issues.

__Voluntariness, Benefits & Risks__: Whether you choose to be involved is up to you. There will be no effect on you or your family. We anticipate that your input will help decisions to be made about how health services are delivered in the future. There is no risk to your health from being in the process. Talking about death and loss can be difficult. We understand that it might make you feel sad or uncomfortable to talk about these things. If the discussions are upsetting or we can stop the discussion or meeting at any time. If you agree to participate, you will also be free to leave the study at any point and for any reason.

__Remuneration & Transport Costs__: You will be reimbursed for your travel for coming to the meetings. We will also provide refreshments during the discussions. Participants also receive a certificate of participation at the end (or when you decide to leave the study- if you choose to).

__Confidentiality and anonymity__: In the reports we may quote parts of the discussion to describe the community's views. Because of this, we cannot guarantee that the conversations will be kept confidential. However, we can guarantee that all the discussions will be private and we will make sure that no-one can be identified in any documents. In the meetings, we will invite you to talk about your opinions and experiences. The information you give is up to you and the meetings will be private. Only those people involved in the process will know whether you are involved. Your identity will not be told to any unauthorized persons; and the discussions will only be shared with people involved in the process. Because the discussions will be in a group, we are not able to ensure the confidentiality of the discussions, but will encourage other participants not to share the identities of other participants with those outside the group. Any information on your identity will be removed before we prepare the reports. Any information that could identify you (name, address etc.) will not be recorded.

CONVENING STAKEHOLDERS

FREQUENCY OF MEETINGS

Ma thulusi lawa ya nga leka manual lowu ya endleriwe ku tirhisiwa hi mavhiki yaku landzelana eka meeting lowu wu ngata endleka vhiki ri n'wana na ri n'wana. Swi kahle ku pfumelela leswaku ti workshop leti ti ngata endleka ku fika eka 5 hi ku landzelana ti fanele ku teka kwalomu ka 2-3 hours na swona ti community stakeholders na ma CHWs va fanela ku tshama eka meeting lowu ku fikela ti awara leti ti hundza. minhlengeletano leyi yi fanele ku pulaniwa xikan'we na ma CHWs na ti community stakeholders.

NUMBERS OF STAKEHOLDERS

Ntlawa wa vanhu wu fanele ku fika kwalomu ka 12 kumbe 16 ya vanhu. Kuya hi nhlokomhaka ya nkoka leyi yi hlawuriweke va ngeneleri vata komberiwa ku rhangela ma n'wana magoza.

INFORMATION

Community stakeholders va fanele ku vulavurisiwa hi foyini kumbe va voniwa Kunene va vulavurisiwa hi nomu, va tlhela va byeliwa hi mintirho leyi yi faneleke ku endliwa na leswaku swi lava va kuma yini eka swona. Lava va swi tsakelaka va nga nyikiwa papila ra xirhambo va tlhela va nyikiwa nkarhi wo ringana ku fikela kwalomu ka 72 hours leswaku va ehleketa loko va swi tsakela ku nghenelela, va nga tlhela va vutisa swivutiso hi foyini kumbe hiku vona munhu leswaku vata kota ku basiseriwa. Ku fanele ku vekiwa nkarhi na siku leri workshop yi ngata endleka hi rona. I swa nkoka leswaku vangeneleri va pfumela ku nghenelela eka maendlelo lawa na swona va twisisa nkongomelo wa maendlelo lawa.

EXPECTATIONS

Tani hi leswi ti community stakeholders ti rhambiwaka eka maendlelo lawa ya community mobilisation, I swa nkoka kuva byela ntiyiso hi mintirho leyi yi ngatava yi endliwa, hi nkarhi lowu va ngatawu teka na ku ncinca loku va nga taku vona hikokwalaho ka maendlelo lawa. I swa nkoka ku pfumelela (a) ku ka ku ngavi na ku titshembha eka ti communities, leti ti twaka onge ku hlanganyela loku ku endliweke na varhangeri va swa rihanyo aku se tshama ku va tirhela kumbe kuva pfuna, ku katsa na (b) swivilelo leswi swi nga kona eka system hi ku angarhela, swi vilelo swo fana na ku kayivela ka ti resources xi kan'we na matirhelo lawa ya nga kona.

Figure 1. Seating participants in a large semicircle supports learning and self-reflection

Figure courtesy of: JHPIEGO, 2020

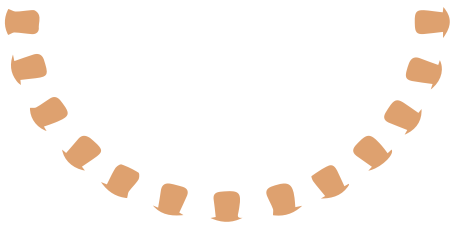

FACILITATION

ACTIVE LISTENING

Ku yingisela hi vukheta I xikili xa kahle lexi munhu a faneleke ku va na xona loko a facilitate mimbhurisano. Ku yingisela swi endla vanhu vatwa onge va twiwa na ku twisisiwa na ku endla leswaku vanhu va hlohloteleka ku vulavula hi ntokoto wa vona, va vulavula hi leswi swi ngale miehleketweni na ku tlhela va vulavula leswi va ti twisaka xiswona hi ndlela yakuka va nga chavi nchumu. Swi kombisa vangheneleri leswaku mavonelo ya vona I ya nkoka loko swita e kaku lulamisa swiphiqo. Ku yingisela swi katsa:

- Pay attention: tirhisa miri wa wena ku kombisa leswaku utekela nhlokweni na ku twisisa. Eka mi ndhavuko yi n'wana leswi swi nga katsa ku pfumela hi nhloko kumbe ku jika u langusa munhu loyi anga le kuvulavuleni;
- Show that you're listening: swi kombisa ku tsakela ka leswi va n'wana va ngale ku swi vuleni. Swi nga katsa ku langusa munhu exikandzeni loko ari karhi a vulavula. Eka ti communities ti n'wana aswi amukeleki ku langusa munhu ematihlweni kumbe exikandzeni loko ku nga seza kuva navu xaka byaku tshembhana exikarhi ka n'wina. U nga yingiseli ntsena ka leswi swi hlayiwaka kambe tlhela u yingisela na leswaku swi hlayiwa njani hi ku langusa miri wa munhu loko ari karhi a vulavula;
- Ask clarifying questions: vutisa swi vutiso ku kombisa leswaku wa swi twisisa. " u nga hlamusela leswaku u fikelele njani eka makumu lawa?" "u switwe kwihi sweleswo?" komisa leswi swi vulavuliwe ku kambisisa leswi swi twisisiweke. kombela feedback;
- No judgement: ti tsundzuxi leswaku mbhurisano awu fanelanga wuva hi mhaka ya wena kumbe wuva hi mhaka ya vanhu lava va pfumelelanaka hi xilo xin'we kumbe ku yima xikan'we. Pfumelela loyi a vulavula leswaku afika ma kumu ka leswi aswi vulavulaka kutani u nga vutisa swi vutiso loko u lava ku basiseriwa.

FACTILITATION DO's AND DON'T's

Swa tsundzuxiwa leswaku eka workshop yi n'wana na yi n'wana kuva na two ya ti facilitators. Eka workshop yi n'wana na yi n'wana, ti facilitators ti fanele ku nyika nkarhi ku langutisisa eka leswi tirheke na leswi swi kalaka swi nga tirhangiki kumbe ku vona leswaku vanga antswisa yini eka ti workshop leti landzelaka.

DO

- Ku titshembha
- Ku miyela u tlhela u titwa kahle hi swona
- Vutisa swi vutiso leswi swi nga laveki nhlamulo ya ina kumbe Ee kambe va kota ku vula vula hi vuenti.
- Rhamba ntlawa wa lava va nga miyela leswaku va ngenelela
- Pfumelela switwi swa wena ku ku komba leswaku u fanele ku endla yini
- Endla leswaku kuva niku lawuleka.

DON'T

- Ku endla onge wa twisisa na kuve a wu twisisi
- Ku vulavula ngopfu
- Ku nyika mavonelo
- Ku tirhisa xiyimo xa wena xa ntirho ku kombakalisa matimba
- Ku tirhisa ntlawa lowu tani hi xilo lexi xi nga ku lulamiselaka swiphiqo swa wena
- ku ahlula va n'wana

TIPS

- Fonela munhu u n'wana na u n'wana workshop yi ngasi sungula
- U nga tirhisi rito "kambe" ti trhisa "na" xikombiso: "u teku....na swona Robert anga pfumelelani na swona"
- Ku vutisa swi vutiso swo tala
- Ku yingisela, ku ehleketa hi vukheta na ku tiyisisa
- Ku langutisisa munhu u n'wana na u n'wana e xikarhi ka meeting
- Ku pfumelela ma vonelo ma n'wana na ma n'wana

IDENTIYING HEALTH CONCERNS

Tool 1/WORKSHOP 1: RANKING AND SCORING

WORKSHOP 1: GUIDE

INTRODUCTION

nhlangano lowu wu fanele ku teka kwalomu ka 2-3 ya ti awara. Ku fanele kuva nati attendance register, register I ya nkoka loko swita ekaku tsala report ya makumu.

I maendlelo yo hluvukisa tiko, hiku aka vutivi eka community naku teka goza eka social and structural determinants of health. Maendlelo lawa ma kongome ku aka vuxaka bya ku tshembana na ku kuma switshuxo eka swiphiqo leswi swi nga kona hiku tirhisana na varhangeri va leka health na vale tikweni.

GROUND RULES

Moya wa ndawu ya hina awu fanelangi wuva lowu wu chuhisaka [Our environment is non-threatening], ku ngavi naku sandzana na xidimokirasi. U n'wana na u n'wana atitwi ari membara ya maendlelo lawa. Hlayani ku tlhela ku vulavuriwa hiti principles ta 4 leti ti nga ka (page 8). Hi team loko hiri hinkwerhu. Hi ta hlengeleta mintokoto leyi hi ngana yona, hi tlhela hi komba swilo leswi hi tsakelaka ku swi kuma.

OUTLINE PROCESS AND ACTIVITIES

- Hita tsakela ku hlangana kan'we hi vhiki, nahi vhiki rin'wana na rin'wana leri ri landzelaka ku fika eka 5 yama vhiki na swona mi nhlengeletano leyi yi ta teka kwalomu ka 2-3 hours.
- Vanhu vanga famba nkarhi u n'wana na u n'wana hi xivangelo xi n'wana na xin'wana.
- Hita vutisa vanhu hi mintokoto leyi vangana yona na mavonelo ya vona mayelana na nhlokomhaka leyi yi hlawuriweke
- Ku suka kwala hi ta sungula ku hlanganisa matwisiselo ya hina na ku endla magoza ekaku lulamisa xiphiqo lexi xi hlawuriweke
- Hita teka hinkwaswu leswi hi swi hlanganiseke na varhangeri vale ka community, kuya hiya tirhisa swona ku vulavurisana na district health system leswaku hita langutisisa, hi tlhela hi pulana xikan'we na vona hiri karhi hi tirhisa vuxokoxoko lebyi hi byi kumeke.
- Hitaswi endla hiku vuyelela, hi tlhela hi langutisa magoza lawa ya tekiweke

WORKSHOP OBJECTIVES

- Ku pfumelelana eka ndhawu na nkarhi wa workshop leyi landzelaka
- Ku komba na ku rhangisa swivilelo leswi swi nga kona eka community
- Ku pfumelelana hi vanhu lava va ngenelelaka eka nhlokomhaka leyi (codesign)
- Ku komba swikili leswi swi kumiweke eka workshop
- Endla nkomiso wa ripoti ya workshop

CHOOSING THE TOPIC

- Landzelela maendlelo ya Tool 1
- Kombela u n'wana wa ma CHW leswaku mava kona eka workshop va tlhela va tsala timhaka ta nkoka leti ku vulavuriwaka hi tona.

CO-DESIGN THE PROCESS

- Ntlawa lowu wu ngava wu yimela community leyi? Ku xoda va mani? Hi nga va nghenisa xana? Leswi swi ngava swi hlamusela yini eka leswi hi swi kumeke? Hi endla yini hi swona leswi?
- Hlamusela rito community tani hi swilo leswi swi vulaka vanhu lava vangana mahanyelo yaku yelana e tikweni.
- Hita tsakela ku tirha na mintlawa yi n'wana leyi yi yimeleke vanhu lava va pfumalaka,xikombiso. Va manana lava ntsongo lava pfumalaka vavanuna? Hi xihi xitsundzuxo xa wena mayelana na mhaka leyi?
- Hi nga ehleketa leswaku lewi swi ngava swi vula yini loko swita eka ku nghenisa vangheneleri vantshwa. Hi nga katsa va mani eka mundzuku?
- Ku vulavurisana na ku pfumelelana hi vanhu lava va nga ta nghenisiwa-record
- nhlengeletano lowu wu nga endleka rini?
- I va mani va nga vuyeriwaka hima endlelo lawa? I va mani lava va khomeke na ku lawula maendlelo?

READING AND VERIFICATION OF GROUP RECORD AND THEN GROUP REFLECTION

FINAL QUESTIONS AND FEEDBACK - DATE/TIME FOR NEXT MEETING

TOOL 1: RANKING AND SCORING

To identify priority health topics of relevance to the community. A list of health priorities is developed during the discussion, after which participants apply scoring in order to identify the topics of highest relevance, using adhesive stickers, beans or sticks. The scoring progresses through at least two rounds with discussion and agreement at the end.

METHOD:

ranking and scoring/multi-dot system

TIME:

30 mins

MATERIAL:

pen and flip chart paper, counters (stones, seeds, markers)

OVERVIEW

Thulu leyi yita pfuna ma CHWs ku komba nhlokomhaka ya xiphiqo lexi xinga kona lexi ku vileriwaka hi xona ka community. Nxaxameto wa ti nhlokomhaka ta swilo leswi rhangisiwaka e mahlweni swa rihanyo wa endliwa hi nkarhi wa mbhurisano, endzhaku ka sweswo vangheneleri va tirhisa ti beans kumbe switikara ku hlawula nhloko mhaka yi n'we leyi va lavaka ku vonana na yona. Maendlelo lawa mata endleka hiku vuyelela kuri karhi ku vulavurisana ku tlhela ku pfumelelana loko hi fika emakumu.

PROCEDURE

1. Kombela vangheneleri leswaku va endla nxaxameto wa swivilelo leswi swi nga kona swa rihanyo eka community va tlhela va tsala na swilaveko swa kona. Va nga swi endla eka flip chart, Flip chart yi nga vekiwa emahlweni ka ntlawa kumbe yi vekiwa ehansi. Loko kuri hiku ntlawa lowu wu katsa vanhu vaku hambana hambana lava va humaka eka swiyimo swaku hambana hambana, mburisano lowu wu nga endliwa. Mi mbhurisano leyi yi nga endliwa hi mintlawa leyi katsaka vanhu va ntsongo ku sivela leswaku kuva naku kucetelana.
2. Loko nxaxameto se wu endliwile, nyika mu ngheneleri u n'wana na u n'wana 3 yama ribye, beans kumbe marker yi n'wana na yi n'wana leyi yi nga kona.va byeli swaku va swi veka etlhelo ka tinhlokomhaka ti nharhu leti ku vileriwaka hi tona leti va ti vonaka leswaku I ta nkoka na swona ti lavaka ku hantla ti lulamisiwa. Loko mungheneleri atwa onge u lava ku hlawula nhlokomhaka yin'we leyi nga ya nkoka eka yena anga yi hlawula ka nharhu. Kombela vangheneleri kuva va bhurisana hi maendlelo ya scoring lawa avari karhi vawa endla.

   Prompts

   - Hi kwihi kuhambana loku ku nga kona eka leswi va ngheneleri va swi rhangisaka emahlweni?
   - U nga ku hlamusela njani ku hambana loku?
   - Leswi swi hi byela yini hiku hambana ka swilaveko loko swita eka ti stakeholders taku hambana hambana?
   - Leswi swina switandzhaku swihi loko swita eka ti programmes ta rihanyu?
   - Leswi swi nga va swi kombisa mavonelo ya vanhu hinkwavo eka community?
3. Vutisa loko vangheneleri va lava ku ncinca tinhlokomhaka leti va ti hlawuleke, loko va swi tsakela va pfumeleli.
4. hlayela swilo leswi aswi tirhisiwa ku scora kutani u tsala ka flip chart. Ntlawa lowu wu salele hi 3 yati nhlokomhaka, langutisa nhlokomhaka leyi yi ngana swilo leswi aku scoriwa hi swona leswi nyingi kutani u tsala ti nhlokomhaka leti eka flip chart.
5. Vutisa vangheneleri leswaku hikokwalaho ka yini va ehleketa leswaku tinhlokomhaka leti nharhu ti lava ku tekeriwa a nhlokweni ngopfu.

   Prompt

   - Sweswi hina list ya swivilelo ku suka eka community, hi yihi leyi ku vileriwaka hi yona ngopfu eka leti nharhu ti nga tsariwa?
6. Eka ti nhlokomhaka letiya tinharhu tekani nkarhi mi hlawula yin'we leyi ku vileriwaka hi yona ngopfu.
7. Tsala u tlhela u kandziyisa mavonelo ya nkoka.
8. hetisisani hi nhlokomhaka leyi yi hlawuriweke kutani mi tsala hansi.

Adapted from: Cavestro, 2003; and Loewenson et al, 2006

WORKSHOP REPORT 1A: GROUP RECORD

	The person who has been recording the most important discussions fills in this record. Facilitator takes digital images of the flip charts with ranking/scoring/multi-dot system. The recorder records queries, comments or areas of debate raised in the discussion below. The scriber reads this record at the end of the session and one of the other participants verifies.
Session recorded by:<br><br>Verified by*:	
1. Purpose and participants	
2. Process and activities (roles and reporting)	
3. Priority health concerns (see Tool 1)	
4. Expanding the process (communities, locations)	
Queries, concerns, areas of debate raised	

* Participant who confirms that the content of this form is a true reflection of the group discussion

Loyi a tsala mimbhurisano ya nkoka ya ntlawa hi yena angata tsala laha hansi. Facilitator u teka swinepe swa flip chart leyi ku nga endleriwa eka yona ranking/scoring/multi-doting system. Nkandziyiso wu kandziyisa swivutiso,ti comment na mi mphikizano mayelana na mbhurisano lowu wu nga laha hansi. Maendlelo lawa ya nga endliwa nakambe eka workshop leyi yi landzelaka.

1. Nkongomelo na vangheneleri
2. Maendlelo na migingiriko
3. Swivilelo swarihanyu swa nkoka
4. Kundlandlamuxa maendlelo
5. Swivutiso, swivilelo na mimphikizano

WORKSHOP REPORT 1B: GROUP REFLECTIONS

	CHW asks another participant in the group to ask these questions and the CHW records the group answers
Session recorded by:<br><br>Verified by*:	
1. What are the main lessons and skills you learned in this workshop?	
2. How will you use the skills obtained in this workshop in future?	
3. What challenges did you experience in this workshop?	
4. Who benefits from this process?	
5. What would you change about the workshops?	
6. Are these skills that you can transfer to other CHWs? What support would you need to do so?	
7. Did the workshop meet the objectives?	

* Participant who confirms that the content of this form is a true reflection of the group discussion

CHW u byela u n'wana wa va ngheneleri eka ntlawa leswaku a vutisa swivutisoleswi kutani CHW a tsala ti nhlamulo leti ti vuriweke hi ntlawa.

1. Hi yihi dyondzo na swikili leswi u swi dyondzeke eka workshop leyi?
2. U ta yi tirhisa njani dyondzo leyi u yi kumeke e ka workshop leyi eka mundzuku?
3. Hi yihi mintlhontlho leyi u hlanganeke na yona eka workshop leyi?
4. I va mani va vuyeriwaka hi maendlelo lawa?
5. I yini lexi u nga xi ncincaka hi ti workshop leti?
6. Leswi ku ngava ku ri swilo leswi u nga swi dyondzisaka va n'wana?
7. Workshop yi ngava yi fikelerile nkongomelo wa yona?

CHW u byela u n'wana wa va ngheneleri eka ntlawa leswaku a vutisa swivutisoleswi kutani CHW a tsala ti nhlamulo leti ti vuriweke hi ntlawa.

1. Hi yihi dyondzo na swikili leswi u swi dyondzeke eka workshop leyi?
2. U ta yi tirhisa njani dyondzo leyi u yi kumeke e ka workshop leyi eka mundzuku?
3. Hi yihi mintlhontlho leyi u hlanganeke na yona eka workshop leyi?
4. I va mani va vuyeriwaka hi maendlelo lawa?
5. I yini lexi u nga xi ncincaka hi ti workshop leti?
6. Leswi ku ngava ku ri swilo leswi u nga swi dyondzisaka va n'wana?
7. Workshop yi ngava yi fikelerile nkongomelo wa yona?

EXAMPLES: RANKING AND SCORING

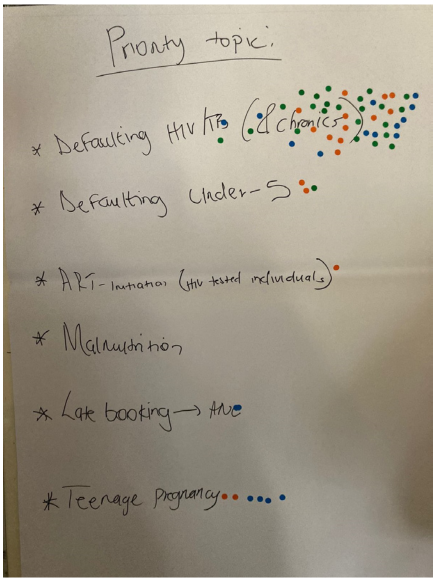

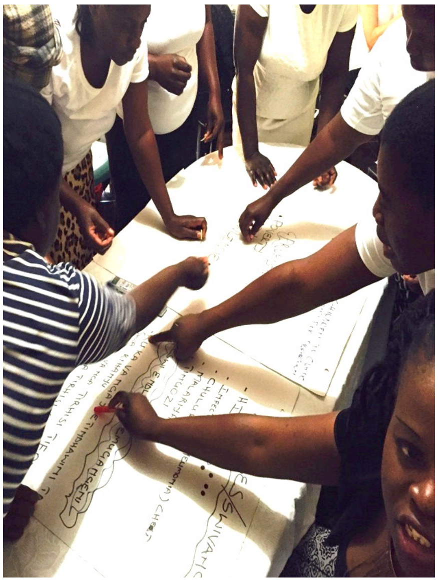

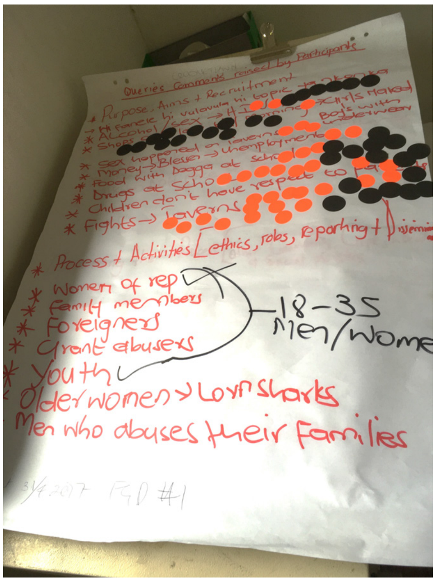

ORGANISING EXPERIENCES

Tool 2 / Workshop 2: PROBLEM TREE

WORKSHOP 2: GUIDE

INTRODUCTION

Workshop leyi yi fanele ku teka kwalomu ka 2-3 yati awara. Ti facilitators ti fanele ku teka swinepe swava ngheneleri na mbhurisano. Ku laveka attendance register, register I ya nkoka loko swita eka ripoti.

GROUND RULES

Ndhawu leyi ku burisaniwaka ka yona yi fanele yi nga chuhisi, yi ngari na swi solo xikan'we na xidimokirasi. U n'wana na u n'wana I membara a tlhela ava part ya maendlelo lawa. Hlayani mitlhela mi vulavula hiti principles ta PAR Principles ku suka eka Handout (page 8). Hinkwerhu hi ntlawa. Hita sungula hiku hlanganisa mintokoto leyi hi ngana yona, hi komba swi laveko na mbuyelo lowu hi tsakelaka kuwu vona.

WORKSHOP OBJECTIVES

- Ku endla ku twisisa ka swivangelo na switandzhaku
- Ku komba swilaveko na kunavela loku hi lavaka kuku vona hi nhlokomhaka leyi yi hlawuriweke [health concern]
- Ku komba swi kili leswi swi kumekeke hi nkarhi wa workshop
- Endla nkomiso wa ripoti ya workshop.

OUTLINE PROCESS AND ACTIVITIES

Xikongomelo xa hina I ku antswisa vutivi eka swilo leswi tekeriwaka nhlokweni. Ku vhakachela nhloko mhaka liya yi nga hlawuriwa. Vangeneleri va rhambiwa ku tsala vutivi bya vona kumbe ntokoto wa vona mayelana na nhlokomhaka leyi yi nga hlawuriwa- mbhurisano wu fanele ku kandziyisiwa.

ORGANISING INTO COLLECTIVE KNOWLEDGE

- Landzelerisa maendlelo ya **Tool 2**
- Kandziyisa mbhurisano.

SUMMARY AND REFLECTIONS

- Sweswi hi hetile mbhurisano wa hina, Mr/s [NAME] va ta hi endlela nkomiso wa leswi ahi burisana hi swona.
- Lowu ku ngava kuri nkomiso wa kahle? Swi kona leswi swi nga pumbiwa?
- Hiri yini hi migingiriko [[activities]? Swi kona leswi hi lavaka ku swi ncinca? Hi swihi swi tirhaka? Hi swihi swi nga tirheki?
- I mani avuyeriwaka hi maendlelo lawa? njani? I mani a lawulaka maendlelo lawa? njani?
- Rhamba ti nhlamulo kumbe swi viko mayela na maendlelo lawa. Vanhu va ehleketa yini mayelana na ku nghenelela?

READING AND VERIFICATION OF GROUP RECORD AND THEN GROUP REFLECTION

FINAL QUESTIONS AND FEEDBACK – siku na nkarhi wa workshop leyi landzelaka

TOOL 2: PROBLEM TREE

INTRODUCTION

This is a tool to understand and 'unpack' nominated topics from different perspectives. Through facilitated discussions using a tree diagram visible to all, participants identify cause-and-effect relationships at various levels from root (tree roots) to intermediary causes (trunk and branches) and consequences and other effects (tree pods), building subjective perspectives into shared accounts through consensus.

Method:

Problem tree (organising causes)

Time:

45mins

Materials:

Copy of the 'problem tree'/ask participants to draw

OBJECTIVE

Twisisa swi vangelo leswi kulu na switandzhaku swa swivilelo leswi swi nga kona mayelana na rihanyu. Antswisa matwisiselo ya swivilelo swarihanyu.

PROCEDURE

Gingiriko lowu wu pfuna vangheneleri ku langutisisa swivangelo swa xivilelo lexi xi hlawuriweke. Wu endla leswaku va kota ku lulamisa xiphiqo lexi hiti ndlela taku hambana hambana.

FOCUS ON PRIORITY HEALTH CONCERN

ti facilitator ti rhamba mavonelo lawa vanhu va ngana wona mayelana na nhlokomhaka leyi yi hlawuriweke, kutani va tsala eka flipchart.

Prompts

- Hi swihi leswi swi tivekaka hi xiphiqo lexi na swi vangelo swa xona?
- Hi yihi mintokoto leyi vanhu va ngana yona eka ntlawa lowu?
- Hi swihi swi kombiso na ku nghenelela loku ku nga endliwaka?
- Vukorhokeri byiri yini?
- Ku ngava kuri na nhlamuselo? Kuvilela loku ku ngava khumba ntlawa lowu hi ndlela yihi? Community yi khumbeka hi ndlela yihi?
- I mani loyi a khumbekaka? Xiphiqo lexi xi ngava xikhumba mintlawa yi n'wana na swona hi ndlela yihi?

List leyi yi endliweke ya kambisisiwa kutani ku vulavuriwa hi yona, vangheneleri va vulavurisana va tlhela va vutisana; ti facilitator ti langutisisa hinkwaswu leswi va swi tsaleke, ku vona leswaku hinkwaswu leswi swi tsariweke I vuxokoxoko bya kahle.

ANALYSE PROBLEM

Hi ku tirhisa xifaniso xa nsinya, vangheneleri va langutisa swivangelo swa xiphiqo lexi.magaji lawa ya ngana matluka ma yimele switandzhaku; magaji lawa ya khomeke hi stem I swivangelo leswi munhu hi yexe ati vangelaka swona; stem xi khome swivangelo swaleka community na le ndyangwini; timintsu tiyimele swivangelo leswi swi endliwaka hi matirhelo yati politiki. Facilitator u tsala hansi leswi a byeriweke swona hi vangheneleri na swona u tsala laha a byeriwaka leswaku a tsala kona.

LOOK AT THE CAUSES AND DISCUSS

Hinkarhi wa mbhurisano, laha ti point ti n'wana ti nga vekiwa kona ka nsinya swi nga ncinca, facilitator anga endla ku ncinca eka diagram ya problem tree.

Prompts:

- Hi swihi swi vangelo leswi community yi nga nghenelelaka eka swona hi ku tirhisa switirhisiwa leswi va ngana swona? Hi ndlela yini?
- Hi swihi leswi ku faneleke ku nghenelelaka va n'wana lava humaka ka district yaleyo?
- Hi vahi lava community yi faneleke ku va hlohlotela ku endla leswaku magoza lawa ya endleka?
- Hi swihi swi lavaka mfumo na varhangeri va leka national?

RECORD

Record on the session record sheet (see overleaf). Facilitator uteka swi faniso swa xifaniso xa problem tree. Facilitator u tsala swi vutiso, kumbe mphikizano lowu endliweke hi nkarhi wa mbhurisano.

Adapted from: Cavestro, 2003 and Loewenson et al, 2006

WORKSHOP REPORT 2A: GROUP RECORD

	The person who has been recording the most important discussions fills in this record. Facilitator takes digital images of the flip charts with ranking/scoring/multi-dot system. The recorder records queries, comments or areas of debate raised in the discussion below. This process may be continued following the workshop. The scribe keeps record of all discussions and reads this record at the start of the next workshops and one of the other participants verifies.
Session recorded by:<br><br>Verified by*:	
1. List words/ features/ experiences to describe [topic]	
2. Suggested shared definition of [topic]	
3. Impacts of topic in this group (preliminary)	
4. Main needs and desired outcomes around [topic]	

* Participant who confirms that the content of this form is a true reflection of the group discussion

WORKSHOP REPORT 2A

Loyi a tsala mimbhurisano ya nkoka ya ntlawa hi yina angata tsala laha hansi. Facilitator u teka swinepe swa flip chart leyi ku nga endleriwa eka yona ranking/scoring/multi-doting system. Nkandziyiso wu kandziyisa swivutiso,ti comment na mi mphikizano mayelana na mbhurisano lowu wu nga laha hansi. Maendlelo lawa ya nga endliwa nakambe eka workshop leyi yi landzelaka.

1. Tsala marito kumbe ntokoto lowu wu hlamuselaka nhlokomhaka leyi kahle.
2. Tinhlamuselo leti vuriweke mayelana na nhlokomhaka leyi.
3. Xitandzhaku/ntshikelelo lowu nhlokomhaka leyi yi nga na wona eka ntlawa lowu.
4. Swi laveko na swilo leswi tsakeriwaka ku kumiwa kumbe mbuyelo lowu wu lavaka ku kumiwa ma yelana na nhlokomhaka leyi.

WORKSHOP REPORT 2B: GROUP REFLECTIONS

	CHW asks another participant in the group to ask these questions and the CHW records the group answers
Session recorded by:<br><br>Verified by*:	
1. What are the main lessons and skills you learned in this workshop?	
2. How will you use the skills obtained in this workshop in future?	
3. What challenges did you experience in this workshop?	
4. Who benefits from this process?	
5. What would you change about the workshops?	
6. Are these skills that you can transfer to other CHWs? What support would you need to do so?	
7. Did the workshop meet the objectives stated in the beginning?	

* Participant who confirms that the content of this form is a true reflection of the group discussion

CHW u byela u n'wana wa va ngheneleri eka ntlawa leswaku a vutisa swivutisoleswi kutani CHW a tsala ti nhlamulo leti ti vuriweke hi ntlawa.

1. Hi yihi dyondzo na swikili leswi u swi dyondzeke eka workshop leyi?
2. U ta yi tirhisa njani dyondzo leyi u yi kumeke e ka workshop leyi eka mundzuku?
3. Hi yihi mintlhontlho leyi u hlanganeke na yona eka workshop leyi?
4. I va mani va vuyeriwaka hi maendlelo lawa?
5. I yini lexi u nga xi ncincaka hi ti workshop leti?
6. Leswi ku ngava ku ri swilo leswi u na swi dyondzisaka va n'wana?
7. Workshop yi ngava yi fikelerile nkongomelo wa yona?

EXAMPLES: PROBLEM TREE

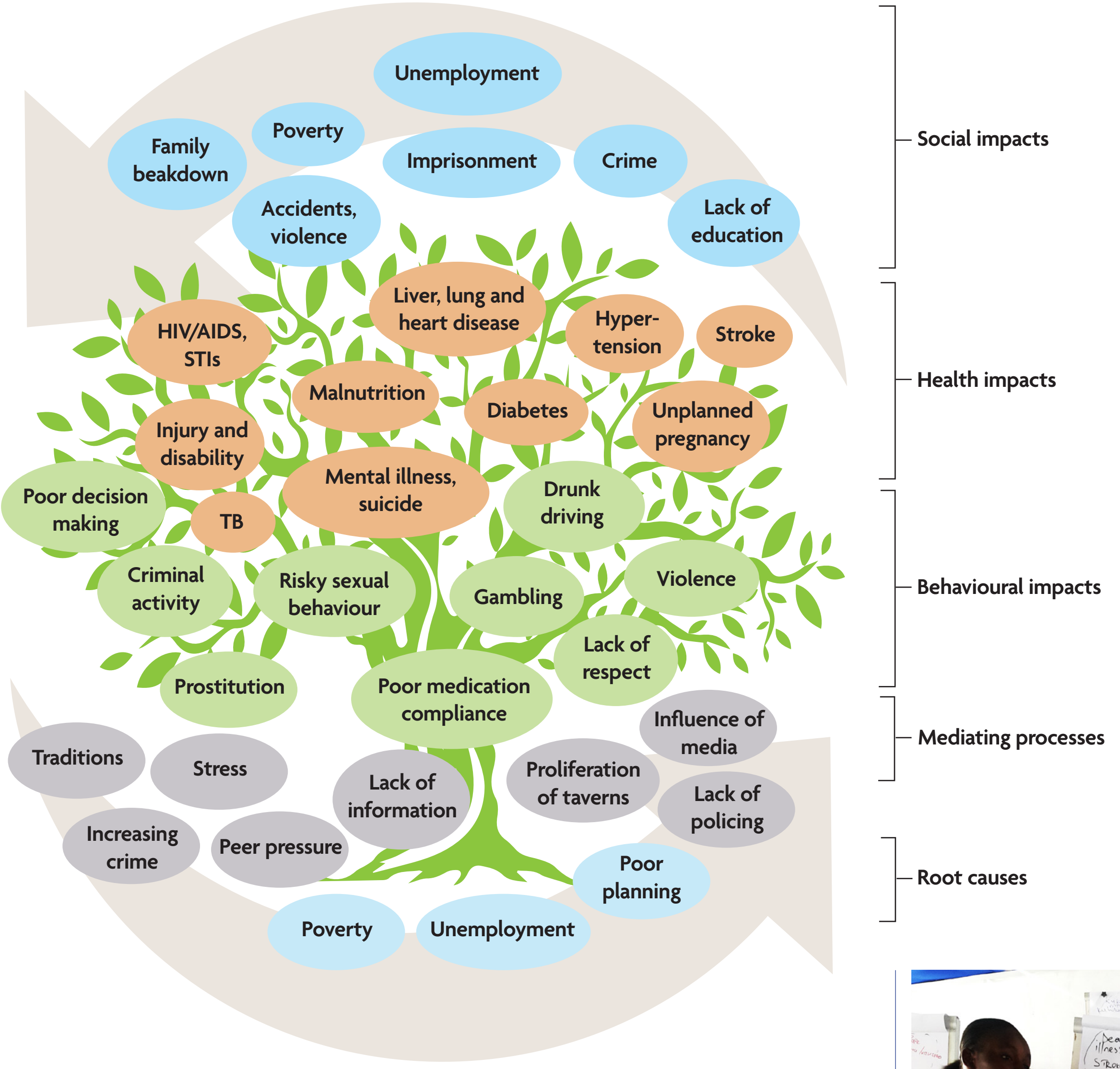

Source: Oladeinde et al, 2020

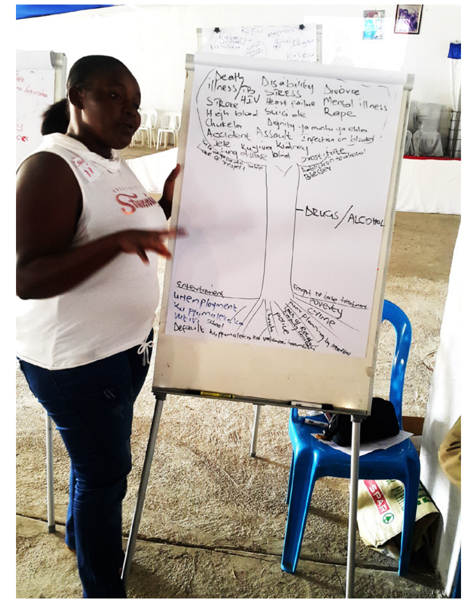

PROBLEM TREE TEMPLATE

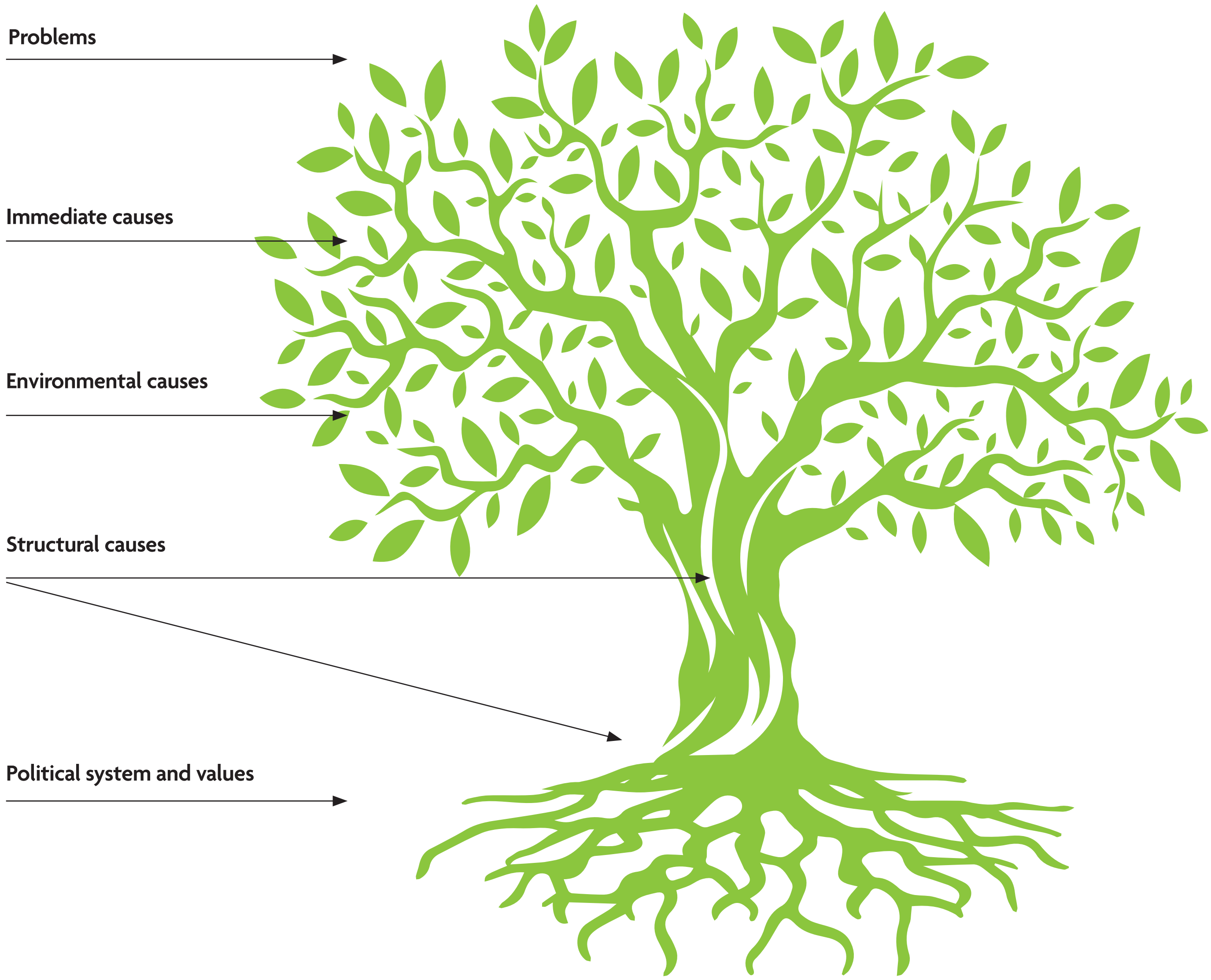

MAPPING ACTORS AND IMPACTS

TOOL 3 / WORKSHOP 3: VENN DIAGRAM

WORKSHOP 3: GUIDE

INTRODUCTION

nhlangano lowu wuta teka 2-3 wa ti awara. Ti facilitators tita teka swinepe swa mbhurisano na vangheneleli. Loko vangheneleli vana xinepe lexi va lavaka kuxi kombisa (see Tool 5) eka van'wana, leswi va nga swi endla hiku swi avela va n'wana hiku tirhisa ti devices. Namuntlha hi lava ku aka kutwisisa/matwisiselo ya hina hi switandzhaku swa xiphiqo lexi.

GROUND RULES

Environment ya hina ayi chavisi, ku ngavi na swisolo na xidimokirasi. U n'wana na u n'wana I membara ya maendlelo lawa. Hlayani mi tlhela mi vulavula hiti PAR Principles ku suka eka Handout (page 8). Hi xilo xin'we hi nkwerhu.lani, hita sungula hi ku hlengeleta mintokoto, hi komba swi laveko na mbuyelo lowu hi tsakelaka kuwu kuma.

WORKSHOP OBJECTIVES

- Ku komba vukorhokeri byaku lulamisa swi laveko swa [xiphiqo xa rihanyu]
- Ku twisisa switandzhaku na ku komba vangwa leri nga kona leri faneleke ku lulamisiwa
- Ku avela swinepe leswi vangheneleri va tsakelaka ku swi kombisa (see Tool 5)
- Ku kombisa vuswikoti lebyi byi kumiweke hi kokwalaho ka workshop
- Ku endla nkomiso wa ripoti ya workshop

OUTLINE PROCESS AND ACTIVITIES

Ti facilitator ti fanele ku kombisa problem tree. Hi endla ku twisisa ko karhi mayelana na xiphiqo lexi.

ADDRESSING THE ISSUES DISCUSSED

Follow **Tool 3:** Venn diagram

SHARING PHOTOS

U n'wana na u n'wana wa vangheneleri leswaku a avela xinepe lexi ku ngata burisaniwa hi xona (see Tool 5).

SUMMARY AND REFLECTIONS

- Sweswi hi hetile ku bhurisana, Mr/s [NAME] vatahi endlela nkomiso wa leswi ahi bhurisana hi swona.
- Lowu ku ngava kuri nkomiso wa kahle?swi ngava swiri kona leswi hi nga swi siya?
- Leswi swi ngava swi vula yini eka mani na swona xihiqo lexi xa rihanyu xi voniwa hi ndlela yihi?
- Leswi swivula yini eka mapulanelo ya swarihanyu, vukorhokeri na leka vatirhi va swarihanyu?

READING AND VERIFICATION OF GROUP RECORD AND THEN GROUP REFLECTION

FINAL QUESTIONS AND FEEDBACK - DATE/TIME FOR NEXT MEETING

TOOL 3: VENN DIAGRAM

This is a tool to understand impacts and actors involved. Collective account developed with Venn diagram made from cardboard circles of different sizes and colours to indicate relationships and interactions between various actors and institutions, identifying internal and external organisations active in the topic and how they related to one another in terms of contact and collaboration. Identify services (health and other) for addressing needs for [health concern]. Identify how services perform in relation to desired outcomes, access, uptake

Method:

Venn diagram (Stakeholder mapping)

Time:

45mins

Materials:

large piece of paper, small coloured pieces of paper, scissors

BACKGROUND:

Venn Diagram yi kombisa ti institutions, organisations, groups na vanhu va nkoka etikweni, xikan'we na mavonelo ya vona leswaku vana nkoka wihi e ka community. Ku tatisa, Venn Diagram yi hlamusela leswaku I va mani vanghenelelaka eka mintlawa leyi kuya hi rimbewu na rifuwo. Venn Diagram yi tlhela yi kombisa leswaku vanhu lava hinkwavo va hlanganyela njani na ku vulavurisana njani.

OBJECTIVES

Ku komba ti organisations/groups/ persons leti gingirikaka eka community [kombisa local area, komba nhlokomhaka ya rihanyu leyi ku vileliwaka hi yona]

Komba leswaku I va mani vanghenelelaka eka local organisations/institutions

Lavisisa leswaku mintlawa leyi hiku hambana ka yona yi hlangana njani loko swita eka ku hlanganyela, ku hangalasa vuxokoxoko na ku nyika vukorhokei.

PROCEDURE

1. Ku tirha hi mintlawa, vangheneleri swilava va vula ti institutions ta swarihanyu leti ti tirhaka eka community ya vona.

   Prompts:

   - Vutisa vangheneleri leswaku hitihi ti organisations/ institutions/ groups leti ti kumekaka eka xitikwana xa vona na swoho hi tihi leti ti kumekaka eka matiko man'wana ti tirhisanaka na vona kumbe leti tiva hlohlotelaka.
   - Endla leswaku va ehleketa na hi letiya ti ntsongo, ku ngari tona leti ti tivekaka leti kulu, xikombiso. neighbourhood committees.

2. Tsala mavito ya ti institutions na vanhu eka flip chart.
3. Nyika vangheneleri ma phephe ya swirhendzevutana swaku va na ti sayizi taku hambana leti ti ngata yimela ti institution/person, loko xirhendzevutana xiri lexi kulu, swi vula leswaku nstitution/munhu loyi una nkoka yaku fika kwini.
4. Byela vangheneleri leswaku va ringanisa ti siyizi ta swi rhendzevutana, ku tani va swi veka kuya hi nkoka wa kona lowu wu yimeleke nkoka wa ntlawa,institution kumbe organization. Vanghneleri va fanele ku tsala vito ra organization,institution kumbe group eka xirhendzevutana lexi va vona swi fanerile ku tsala eka xona. Loko swi fika lani va ngheneleri va ku tala va nga kumiwa, leswi swi lava va tsariwa eka list kutani vata fakiwa eka swirhendzevutana leswi faneleke.

   Prompts:

   - Hitihi ti ndlela taku pfuna hi xiphiqo xa rihanyu leti ti nga kona?
   - Hi yihi mintlawa ya loacal leyi yi nga organised?
   - Mintlawa ya ti politiki yi ngava yiri kona?
   - I va mani va endlaka swiboho swa nkoka e tikweni?
   - Va ngava va ri kona vanhu lava va vilelaka/va tirhaka hi swa tipolitiki, swi fuwo etc? I va mani na swona va va contribute njani?

5. Byela va ngheneleri ku veka swirhendzevutana ehenhla ka phephe leri kulu, kutani va kombisa vuxaka na ku hlangana loku ku nga kona – ku hlangana ka swirhendzevutana swi kombisa ku hlanganyela loku ku nga kona exikarhi ka ti institutions kumbe ti organizations, ku hambana ka swirhendzevutana swi kombisa leswaku akuna ku hlangana exikarhi ka ti instituions kumbe ti organizations teleto, kumbe aswi tirhi hi swilo swaku yelana. Vangheneleri vanga tsongahata ti sayizi kumbe va endla arrangement yi n'wana tani hi leswi vona va swi vonisaka xi swona.

6. Loko va ngheneleri vaha endla diagram, va vutisi leswaku hikokwalaho ka yini va endla swilo swo karhi.

   Prompts:

   - Hikokwalaho ka yini institution leyi yiri kule ka leti n'wana?
   - Ti insttutions leti ta tlulelana – hi yihi migingiriko leyi va avelanaka ha yona?
   - Tsala leswi va swi vulaka.

7. Emakumu endla leswi landzelaka

   - Kombisa leswaku ti institutions leti ti hlangana hi ndlela yihi.
   - Langusa loko vanhu vo karhi xikombiso,vava sati,vanhu lava va nga tirhiki,ti migrants kumbe lava va pfumalaka etc, va ngava va humesiwile leswaku va nga ngheneleri ka ti institutions to karhi. Nyika swi vangelo swa leswaku hikokwalaho ka yini va nga yimeriwi na swona vati twisa ku yini hi swona.

Record on the session record sheet (see overleaf). Facilitator u teka swinepe swa mbhurisano, a tlhela a tsala hansi swi vutiso na mphikizano lowu wu nga endleka.

Adapted from: Cavestro, 2003 and Loewenson et al, 2006

WORKSHOP REPORT 3A: GROUP RECORD

	The person who has been recording the most important discussions fills in this record Facilitator takes digital images of the flip charts with ranking/scoring/multi-dot system. The scriber records queries, comments or areas of debate raised in the discussion below. This process may be continued following the workshop. The scribe keeps record of all discussions and reads this record at the start of the next workshops and one of the other participants verifies.
Session recorded by:<br><br>Verified by*:	
1. Impact of this topic in the group (build in preliminary)	
2. Suggested shared definition of impacts	
3. Needs: identify priority gaps/ weaknesses to be addressed and strengths to be reinforced	
4. Main actors who can address these	

* Participant who confirms that the content of this form is a true reflection of the group discussion

Loyi a tsala mimbhurisano ya nkoka ya ntlawa hi yena angata tsala laha hansi. Facilitator u teka swinepe swa flip chart leyi ku nga endleriwa eka yona ranking/scoring/multi-doting system. Nkandziyiso wu kandziyisa swivutiso,ti comment na mi mphikizano mayelana na mbhurisano lowu wu nga laha hansi. Maendlelo lawa ya nga endliwa nakambe eka workshop leyi yi landzelaka.

- Ntshikelelo lowu nhlokomhaka leyi yi ngana wona eka ntlawa lowu
- Mavonelo ya ti nhlamuselo leti nyikiweke mayelana na nhlokomhaka
- Swilaveko: kombisa mavangwa na ku pfumala matimba loku ku nga kona ku lavaka ku lulamisiwa na matimba lawa ya faneleke ku fakiwa.
- Lava va ngata nghenelela ku lulamisa swilo leswi.

WORKSHOP REPORT 3B: GROUP REFLECTIONS

	CHW asks another participant in the group to ask these questions and the CHW records the group answers
Session recorded by:<br><br>Verified by*:	
1. What are the main lessons and skills you learned in this workshop?	
2. How will you use the skills obtained in this workshop in future?	
3. What challenges did you experience in this workshop?	
4. Who benefits from this process?	
5. What would you change about the workshops?	
6. Are these skills that you can transfer to other CHWs? What support would you need to do so?	
7. Did the workshop meet the objectives?	

* Participant who confirms that the content of this form is a true reflection of the group discussion

CHW u byela u n'wana wa va ngheneleri eka ntlawa leswaku a vutisa swivutisoleswi kutani CHW a tsala ti nhlamulo leti ti vuriweke hi ntlawa.

1. Hi yihi dyondzo na swikili leswi u swi dyondzeke eka workshop leyi?
2. U ta yi tirhisa njani dyondzo leyi u yi kumeke e ka workshop leyi eka mundzuku?
3. Hi yihi mintlhontlho leyi u hlanganeke na yona eka workshop leyi?
4. I va mani va vuyeriwaka hi maendlelo lawa?
5. I yini lexi u nga xi ncincaka hi ti workshop leti?
6. Leswi ku ngava ku ri swilo leswi u na swi dyondzisaka va n'wana?
7. Workshop yi ngava yi fikelerile nkongomelo wa yona?

EXAMPLES: VENN DIAGRAM

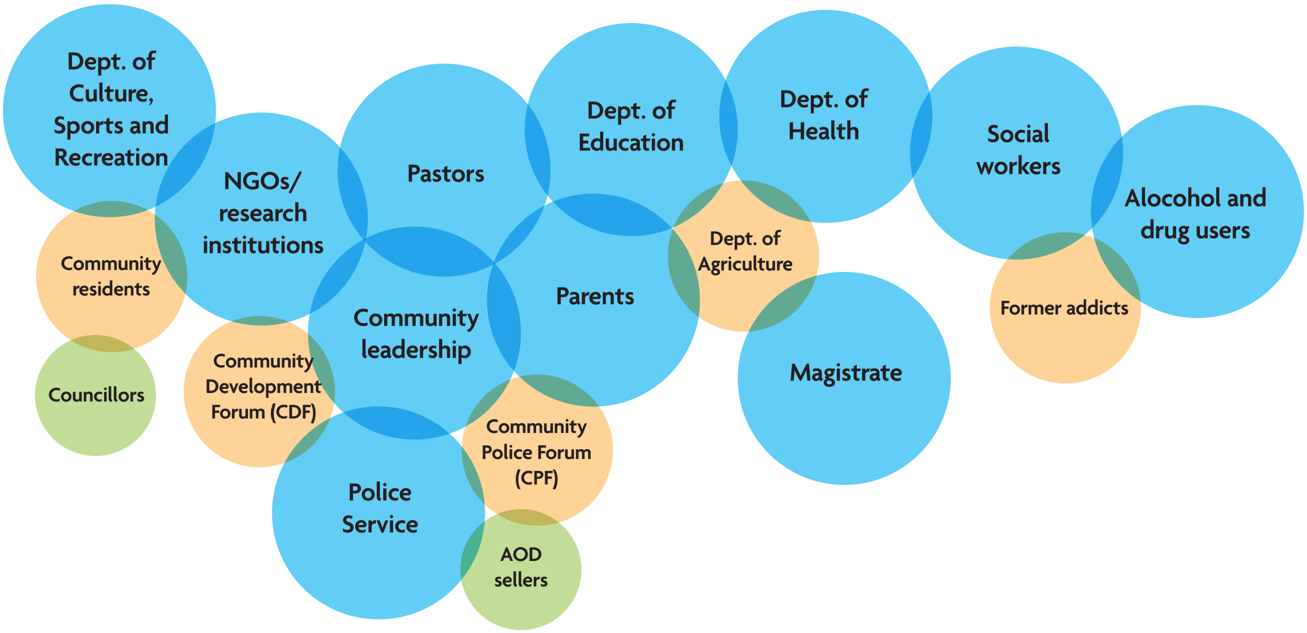

Key

- Most important actors/ institutions (blue)
- Important actors/ institutions (orange)
- Less important actors/ institutions (green)

Source: Oladeinde et al, 2020

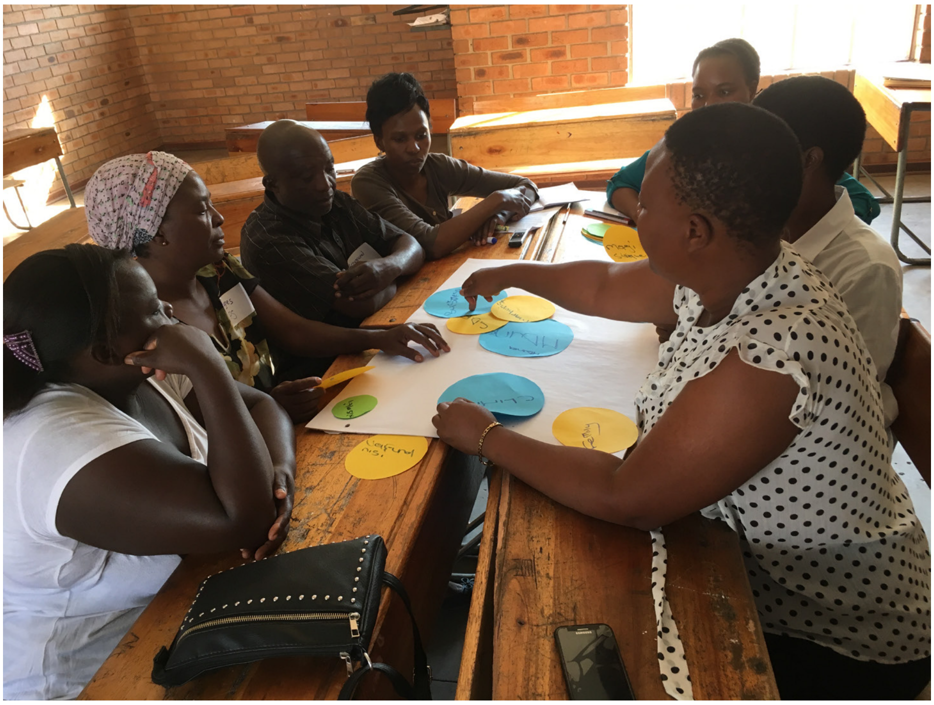

PATHWAYS TOWARDS CHANGE
TOOL 4 / WORKSHOP 4: ACTION PATHWAY

WORKSHOP 4: GUIDE

INTRODUCTION

Nhlangano lowu wuta teka kwalomu ka ti awara taku fika ka 2-3. hi endle problem tree na Venn diagram ku suka eka workshops liya ayiri kona yale ndzhaku, namuntlha hita eka lexi vuriwaka pathway laha hi ngata endla magoza yaku lulamisa xiphiqo lexi xi nga kombisiwa.

GROUND RULES

Ndhawu ya hina yi fanele kuva yi nga chuhisi/ku chavisa, yi ngavi na swisolo xikan'we na xidimokirasi. U n'wana na u n'wana I xirho xa maendlelo lawa. Hlayani mi tlhela miburisana hi ti PAR Principles ku suka eka Handout (page 8). Loko hiri swin'we hi ntlawa/xilo xin'we. lani, hita sungula ku hlanganisela mintokoto ya hina, hi kombisa swilaveko na mbuyelo lowu hi tsakelak ku wu vona.

WORKSHOP OBJECTIVES

- Ku endla magoza leswaku hita lulamisa xiphiqo lexi hi ngana xona
- Vangheneleri lava va tsakelaka ku kombisa swinepe vanga endla
- Ku kombisa vuswikoti lebyi kumiweke eka workshop
- Ku endla nkomiso wa ripoti ya workshop

OUTLINE PROCESS AND ACTIVITIES

Ti facilitator ti ta kombisa problem tree na Venn diagram ku suka eka nhlangano lowu nga hundza. Ti facilitator ti vhakachela swilaveko na mbuyelo mayelana na xiphiqo hi ku langutisa problem tree.
Ti facilitatorti langutisa vangheneleri, ti institutions na vukorhokeri ku suka eka ti institutions ti n'wana ta rihanyu hi ku langutisa Venn diagram. Hi migingiriko leyi ku suka kwala hita endla goza rin'we leri ri ngata tekiwa ku lulamisa xiphiqo lexi.

PRIORITISING ACTIONS

Follow Tool 4: Action Pathways

Actions are listed and ranked.

SHARING PHOTOS

Vangheneleri va kombisa xinepe xin'we kutani va bula hi xona (see Tool 5).

SUMMARY AND REFLECTIONS

- Sweswi hi hetile ku bhurisana, Mr/s [NAME] vatahi endlela nkomiso wambhurisano wa hina.
- Nkomiso lowu wu ngava wuri kahle? Swi ngava swiri kona leswi nga siyiwa?
- Leswi swi ngava swi vula yini eka mani na leswaku xiphiqo lexi xivoniwa hi ndlela yini?
- Swi ngava swi vula yini loko swita eka mapulanelo, vukorhokeri na lekava tirhi va swarihanyu?

READING AND VERIFICATION OF GROUP RECORD AND THEN GROUP REFLECTION

FINAL QUESTIONS AND FEEDBACK - DATE/TIME FOR NEXT MEETING

TOOL 4: ACTION PATHWAYS

This is a tool to articulate overall goal(s) to address the issues identified and visualise and depict stepwise actions and actors to achieve these. The action pathway was collectively developed to represent moving towards a desired goal via a series of interconnected steps and events

Method:

Action pathways (ranking and scoring/ multi-dot system)

Time:

45mins

Materials:

pen, flip chart, counters (stones, seeds, adhesive markers)

PROCEDURE

Ngingiriko lowu wu endleliwe leswaku ku kotiwa ku pulaniwa swilo leswi swi ngata teka nkarhi wo leha swahari kona –ku tirhisa diagram leyi ku lulamisa swi phiqo hi ndlela yo olova.

OBJECTIVES

- Langutisisa problem tree kutani mi endla magoza nava ngheneleri ka venn diagram
- Endla magoza hiku tirhisa action pathways

PROCEDURE

1. Kombela vangheneleri ku endla magoza lawa ya nga endlekaka eka community, hiku langutisa Problem Tree na Venn diagram leyi endliweke. Va nga endla leswi eka flip chart, yi nga vekiwa mahlweni ka ntlawa kumbe ehansi.

   Prompts

   - Hi swihi swivangelo leswi vangheneleri va nga ngenelelaka ka swona hi ku tirhisa switirhisiwa leswi va ngana swona? njani?
   - Hi swihi swi lavaka ku nghenelelaka hi va n'wana lava kumekaka ka district yeleyo?
   - Hi vahi lava community yi lavaka kuva hlohlotelaka ku endla magoza lawa ya endleka?
   - Hi swihi swilavaka mfumo kuva wu swi endla kumbe ti institutions tin'wana?
   - Magoza lawa ya nga endleka rini?
   - Hi ngawa landzelerisa njani?
   - Hi ta swi tivisa ku yini loko ya endlekile?
   - Hi xihi xiphemu xa DoH? Hi rihi rito ra community?

2. Loko list yi endliwile, nyika mungheneleri u n'wana na u n'wana swiribyana swi nharhu, ti beans kumbe marker yi n'wana na yi n'wana leyi yi nga kona. Va kombeli leswaku va swi veka e tlhelo ka magoza manharhu lawa va vonaka onge I ya nkoka ngopfu eka vona na swona swi lava ya hantla ya endliwa. Kombela va ngheneleri va bhurisana na ku vutisana hi maendlelo lawa va nga wa endla ya scoring. Tlhelani mi endla maendlelo ya scoring nakambe: balela magoza ti voti leti ti nga endliwa kutani u tsala ka flip chart paper. Ntlawa lowu se wuna list ya magoza lawa ya tekeriwaka nhlokweni. Tsala magoza lawa eka flip chart paper yi n'wana.

3. Vutisa vangheneleri leswaku hikokwalaho ka yini va ehleketa leswaku magoza lawa ya fanele ku tekeriwa enhlokweni.

4. Action pathway: magoza lawa ya ngatava na mbuyelo loko swita eka mundzuku. Siya diagram ya action pathway eka flip chart paper leswaku yi ta tirhisiwa kambe loko yi laveka.

   - Nyika ntlawa flipchart paper, pens, and pieces of A5 paper with the words "action, actor, when and monitored" written on them (see example on the next page).
   - Tsala magoza (vangheneleri, rini na leswaku njani) ku suka eka xitepu lexi endliweke eka A5 paper, vanga famba famba eka mintlawa ya vona ku fikelela laha vanga ta pfumelelana hi magoza lawa va lavaka kuwa teka.
   - Swi endli swi twala leswaku ahi swa nkoka ku ta na goza leri pfumalaka xihoxo kumbe leri ri nga kahle ngopfu (va nga tlhela va hlawula kuka va nga faki magoza lawa ava ma tsarile ka A5).
   - Loko se va endlile action pathway, va kombeli va tsala exikarhi ka magoza na mbuyelo.

Prompts

- Hi wihi mbuyelo lowu wu ngata huma emaheteleleni? Ku ngava kuri swilo leswi hi vonaka onge swi nga endleka leswi?
- Magoza lawa ya nga leka ti sticky notes ya pfunetakwini ku fikelela magoza lawa?
- Hi nga endla magoza lawa hi ntlawa?
- Hi wahi maendlelo ya kona?
- Hi nga pfumelelana hi goza leri hi faneleke kuri endla?
- Goza leri ri nghenelela njani ka leri n'wana?
- Hi ngawa landzelerisa njani?
- Hi taswi tivisa ku yini loko se ya endliwile?

Ku landzelerisa magoza I swa nkoka ngopfu loko swita eka participaty methods leswaku u ta kota ku dyondza swi n'wanyana.vuxokoxoko byi n'wana byi nga kumeka kwala: **https://www.tarsc.org/publications/documents/MCV%20Implementers%20Resource%202021%20for%20web.pdf**

5. Hetisisa magoza kutani u tsala hansi

Adapted from: Cavestro, 2003; Loewenson et al, 2006; Convention on Wetlands, 2008

WORKSHOP REPORT 4A: GROUP RECORD

<table>
<tr><td></td><td colspan="3">The person who has been recording the most important discussions fills in this record Facilitator takes digital images of the flip charts with ranking/scoring/multi-dot system. The scriber records queries, comments or areas of debate raised in the discussion below. . This process may be continued following the workshop. The scribe keeps record of all discussions and reads this record at the start of the next workshops and one of the other participants verifies.</td></tr>
<tr><td>Session recorded by:

Verified by*:</td><td colspan="3"></td></tr>
<tr><td>1. What are the main features, needs and desired outcomes around [topic]</td><td colspan="3"></td></tr>
<tr><td>2. What are the actions required, how can these be achieved, by who and when?</td><td colspan="3"></td></tr>
<tr><td>3. Can we organise these actions?</td><td colspan="3"></td></tr>
<tr><td>How</td><td>Who</td><td>When</td><td>Monitoring</td></tr>
<tr><td></td><td></td><td></td><td></td></tr>
</table>

* Participant who confirms that the content of this form is a true reflection of the group discussion

Loyi a tsala mimbhurisano ya nkoka ya ntlawa hi yena angata tsala laha hansi. Facilitator u teka swinepe swa flip chart leyi ku nga endleriwa eka yona ranking/scoring/multi-doting system. Nkandziyiso wu kandziyisa swivutiso,ti comment na mi mphikizano mayelana na mbhurisano lowu wu nga laha hansi. Maendlelo lawa ya nga endliwa nakambe eka workshop leyi yi landzelaka.

1. Hi swihi swilaveko na mbuyelo lowu hi lavaka ku wu kuma mayelana na nhlokomhaka leyi
2. Hi wahi magoza lawa ya faneleke ku tekiwa ku lulamisa swilo leswi? Ya fanele ku endliwa hi va mani na swona rini?
3. Hi wa hlelisa ku yini magoza lawa?

Njani Mani Rini Kulandzelela

WORKSHOP REPORT 4B: GROUP REFLECTIONS

	CHW asks another participant in the group to ask these questions and the CHW records the group answers
Session recorded by:<br><br>Verified by*:	
1. What are the main lessons and skills you learned in this workshop?	
2. How will you use the skills obtained in this workshop in future?	
3. What challenges did you experience in this workshop?	
4. Who benefits from this process?	
5. What would you change about the workshops?	
6. Are these skills that you can transfer to other CHWs? What support would you need to do so?	
7. Did the workshop meet the objectives?	

* Participant who confirms that the content of this form is a true reflection of the group discussion

CHW u byela u n'wana wa va ngheneleri eka ntlawa leswaku a vutisa swivutisoleswi kutani CHW a tsala ti nhlamulo leti ti vuriweke hi ntlawa.

1. Hi yihi dyondzo na swikili leswi u swi dyondzeke eka workshop leyi?
2. U ta yi tirhisa njani dyondzo leyi u yi kumeke e ka workshop leyi eka mundzuku?
3. Hi yihi mintlhontlho leyi u hlanganeke na yona eka workshop leyi?
4. I va mani va vuyeriwaka hi maendlelo lawa?
5. I yini lexi u nga xi ncincaka hi ti workshop leti?
6. Leswi ku ngava ku ri swilo leswi u na swi dyondzisaka va n'wana?
7. Workshop yi ngava yi fikelerile nkongomelo wa yona?

EXAMPLES: ACTION PATHWAYS

Overall goal: reduce rates of AOD abuse

Supply reduction	Demand reduction			Harm reduction
**Action:** Reduce opening hours in taverns, ban sale of alcohol to children<br>**Actor:** Police<br>**When:** Daily<br>**Monitoring:** Taverns close earlier	**Action:** Organise awareness campaigns working with Induna<br>**Actor:** Councillor<br>**When:** Weekly<br>**Monitoring:** Improved relationships between leaders	**Action:** Teach people about the dangers of AOD abuse<br>**Actor:** DoH/clinics<br>**When:** Daily<br>**Monitoring:** Register of people taught	**Action:** Offer recreational activities (drop-in centre)<br>**Actor:** DSD<br>**When:** Daily<br>**Monitoring:** Number of youths attending after schools	**Action:** Ensure counselling offered for addicts and families<br>**Actor:** Social workers<br>**When:** Once or twice weekly<br>**Monitoring:** Behaviour change among addicts
**Action:** Ensure drug dealers are arrested<br>**Actor:** Induna, Police and CDF<br>**When:** Weekly<br>**Monitoring:** Reports of people arrested	**Action:** Ensure children do not use AOD, strict house rules<br>**Actor:** Parents<br>**When:** Daily<br>**Monitoring:** Search school bags and rooms to make sure no AOD	**Action:** Raise awareness about AOD harms with health education<br>**Actor:** DoH (district)<br>**When:** Monthly<br>**Monitoring:** Attendance register	**Action:** Create sports facilities<br>**Actor:** Dept. Culture, Sports and Recreation<br>**When:** Before end 2018<br>**Monitoring:** Visible sports grounds	
**Action:** Give long sentences to drug dealers<br>**Actor:** Magistrates<br>**When:** Consistently and as necessary<br>**Monitoring:** Recorded sentences	**Action:** Pray and teach word of God<br>**Actor:** Pastors<br>**When:** Every church service<br>**Monitoring:** Increased number of church goers	**Action:** Develop curriculum on the dangers of AOD abuse<br>**Actor:** DoE/national<br>**When:** Before end 2018<br>**Monitoring:** Subject in the 2018 curriculum	**Action:** Issue seeds to residents to develop community gardens<br>**Actor:** DoH<br>**When:** Every season<br>**Monitoring:** Regular visits to community gardens	

Source: Oladeinde et al, 2020

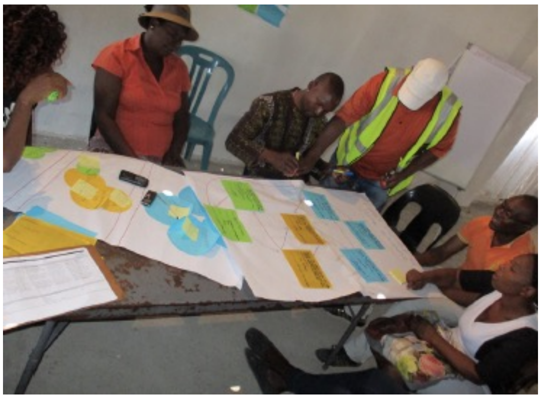

COLLECTING AND ANALYSING VISUAL DATA

TOOL 5: PHOTOVOICE

This tool can be applied where resources and time allow to visually convey lived experience. Participants given basic training in photography, ethics and digital cameras or using smart phones to take photographs illustrating the topic or condition as it existed in the physical environments. Photographs presented and discussed in meetings, and captions developed to describe what images conveyed.

METHOD:

Photovoice

MATERIAL:

digital cameras or smart phones, notepads and pens

OVERVIEW

Ku kombisa mintokoto leyi vanhu va ngana yona. Vangheneleri va nyikiwa dyondzo yi ntsongo hiku teka swinepe, va tirhisa swenepe ku hlamusela swilo leswi endlekaka leswi fambelanaka na nhlokomhaka leyi yi hlawuliweke. Swinepe swa kombisiwa eka nhlangano kutani ku vulavuriwa ku tsariwa leswaku swinepe leswi swi ngava swi vula yini.

WHAT IS PHOTOVOICE?

Photovoice [ku endla marito hiku tihrisa swinepe] i method lowu pfunaka leswaku vanhu va tirhisa swinepe ku hlamusela xitori xa vona. Photovoice yi pfumela leswaku a kuna loyi a faneleke ku tsoniwa nkarhi wova a vulavula na leswaku a ta twiwa.

BASICS OF PHOTOGRAPHY

Ti stakeholders tita dyondzisiwa hiku teka swinepe, va ta kombiwa leswaku va fanele ku endlisa ku yini loko va lava ku tirhisa camera ku teka swinepe.

USING YOUR PHONE TO TAKE AND SEND PROFESSIONAL PICTURES

Mbhurisano lowu fambelanaka na leswaku I va mani vanga na ti email, WhatsApp kumbe tindlela ti n'wana taku bhurisana ku tlhela ku bhuriwa hi ndlela yo kota ku rhumela swifaniso leswi swi ngana mpfumelelo ku suka eka vanhu lava va ngale ka swona leswaku va nga swi rhumelela ka ti devices tin'wana.

EXERCISES

Ti stakeholders ti komberiwa ku va ti endla mintlawa leswaku tiya teka swinepe swinharhu va tlhela va vuya endzhaku ka 15 mins kuta kombisa naku bhurisana. Swifaniso leswi swi ngava swiri swamisava, vanhu/swinepe, ntlhaveko, etc. va vuya eka ntlawa kuta bula hi swinepe leswi swi tekiweke. Ku teka tinotsi ti ntsongo hi swinepe leswi tekiweke, loyi anga teka swinepe, I xa yini, xi hlamusela yini.

SOME GROUND RULES

Vangheneleri va fanele ku hlamusela vanhu lava va vava tekaka swinepe leswaku hikokwalaho ka yini vava teka swinepe na swona swinepe leswi va lava ku swi tirhisa kwini; KOMBELA MPFUMELELO U NGASI TEKA MUNHU XINEPE. Va fanele ku nyikiwa papila ra mpfumelelo loko swikoteka. Loko swi nga koteki, ku fanele ku tirhisiwa marito ku pfumelelana. U n'wana na u n'wana una pfumelelo ya ndhawu yova ari wexe na leswaku a tivisiwa leswaku yena I fanisiwa na yini kumbe xiyimo xa yena xi fananisiwa na yini.

Loko vanhu va vonaka ka swinepe, ku fanele ku kombeliwa mpfumelelano. Ku tatisa kwalano, va fanele ku ehleketeleriwa ngopfu ngopfu ekaku va sirhelela eka swiyimo swa kuka swi nga tsakisi. Leswi swi nga endliwa hiku endla leswaku swikandza swa vona swi endliwa leswaku swi nga vonaki na ku endla leswaku swinepe leswi swi nga tirhi eka local area ya laha swi nga tekiwa yona.

Further ethical guidance for the use of Photovoice is available it: **https://photovoice.org/wp-content/uploads/2017/05/Ethical-Statement.pdf**

Adapted from: Photovoice, 2015

EXAMPLES: VISUAL EVIDENCE (PROCESS)

Community stakeholder loyi a ngale ku vulavuleni ari karhi a tirhisa xinepe lexi anga xikuma hi mpfumelelo

“hi ku vulavula hi ku tirhisa swinepe leswi kumiweke hi maendlelo lawa,ti community stakeholders ti aka vuswikoti bya tona loko swita ekaku vulavula entshungwini, ekaku vulavulavula eka mintlawa xikan’we na ku aka vuxaka”

Photographer: Denny Mabetha, VAPAR coordinator

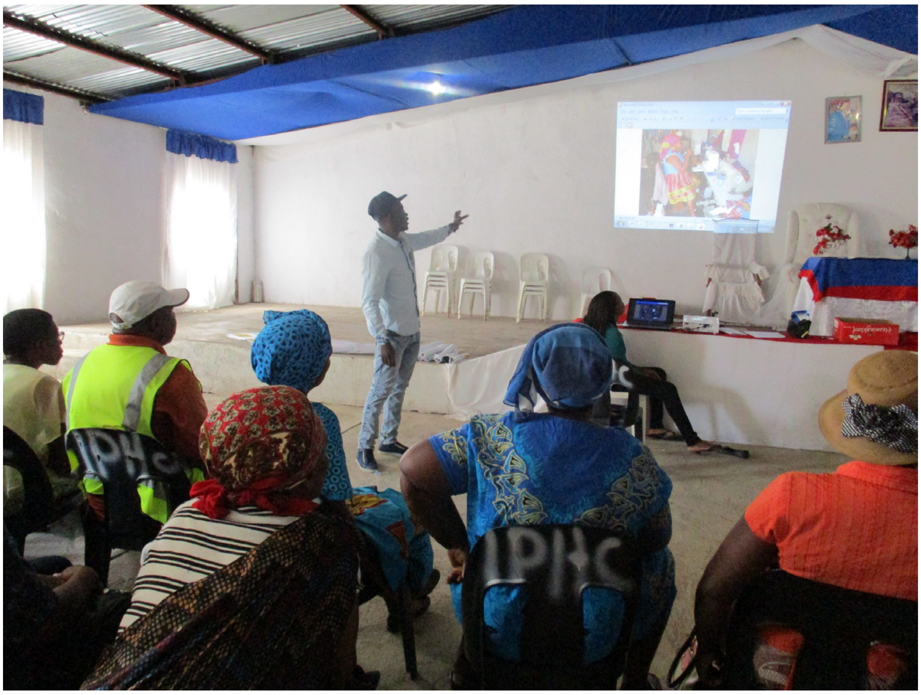

EXAMPLES: VISUAL EVIDENCE

Examples of visual evidence on alcohol and drug abuse, with captions developed by community stakeholders

ALCOHOL AND DRUGS - THE STRESS RELIEVERS

Wanuna loyi anga le ku cineni a tlhela a yimbelela endzhaku ka loko atava a n'we byalwa na ku dzaha. Byala na swidzidziharisi swi voniwa tani hi swilo leswi susaka xitirese eka vanhu lava va hanyaka eka swiyimo swaku tika. Kuya hi vangheneleri, byala byiva endla va rivala swiphiqo swa vona byi tlhela byiva pfuna ku kota kuhanya kahle eka siku ra mundzuku.

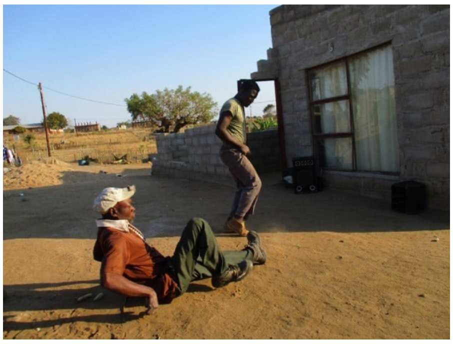

Photographer: Delight Zitha / VAPAR learning platform

YOUTH DRINKING AND DRIVING

Wanuna lontsongo loyi angale ka xidirayivele xa movha na vusiku na bodlhela ra byala kuve ka voko ri n'wana u khome selulafoyini. Vanhu vale tikweni va vula leswaku leswi hi swona swi endlaka leswaku kuva na mafu ya ku tala na vugono, swi tlhela swi vanga na tingozi etindleleni.

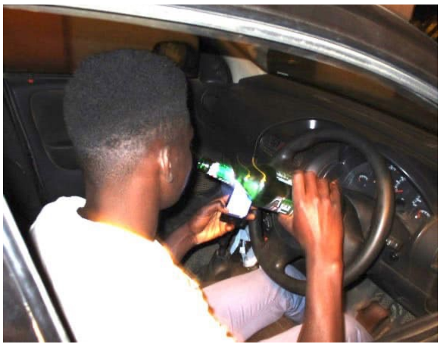

Photographer: Cornright Zitha / VAPAR learning platform

ALCOHOL ABUSE IN OLDER PEOPLE

Wansati lo nkululoyi anga tlela ehansi endzhaku ka loko a tave a n'we mabodlhela ma ngari ma ngani ya byalwa. Xinepe lexi xi tekiwe hi ti awara to sungula ta siku leswi swi kombisaka leswaku byala byi n'wiwa ngopfu eka community: aswi bihangi kuva munhu a n'wa byala hi nkarhi u n'wana na u n'wana wa siku.

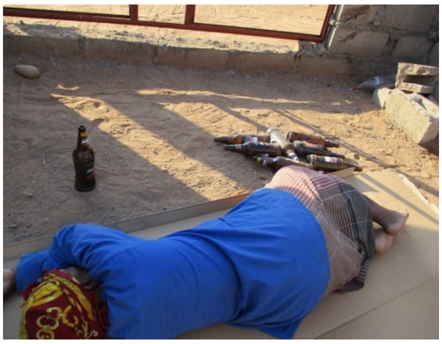

Photographer: Ennie Khoza / VAPAR learning platform

NEXT STEPS: GIVING COMMUNITY VOICE TEETH

NEXT STEPS

Swi n'wana swa swivilelo leswi swi ripotiweke hi ma CHWs ma n'wana I ku pfumala kaku tshembhana exikarhi ka vona na ti communities, na ku pfumala ka switirhisiwa.hambi swiri tano ma CHWs ma endla swilo swankoka eka ti communities, va tiveka tani hi vanhu lava va tirhaka ntsena hi HIV, ntirho wa vona wa leswi n'wana va swi endlaka eka community awu tiveki.

DEVELOPING TRUST RELATIONSHIPS IN COMMUNITIES

Ku tshamela ku vulavula na ku hlanganyela hi ndlela leyi yi ngana ku xiximana swi aka vuxaka bya kahle exikarhi ka ma CHWs na ti communities. Vanhu va ku tala va leka community ava swi tivi swilo swo fana na ti community feedback meetings, leadership meetings na ti ndhawu tin'wana laha ti stakeholders ti nhlanganyelaka kona ku burisana hi swi phiqo leswi endlekaka etikweni. Kuna tindlela to tala leti ti nga tirhisiwaka ku tlakusa rito ra community na ku seketela vumbhoni lebyi byi endliweke hi maendlelo ya participatory methods.

CONNECTING TO RESEARCHERS

VAPAR yi kumeka MRC/Wits-Agincourt Unit's Health and socio-demographic surveillance system, leyi yi tisaka vuxokoxoko lebyi fambelanaka na swivilelo leswi vuriweke. VAPAR researchers va seketela maendlelo lawa leswaku ya kota ku pfuna eka ku hlanganyela, na ku teka hinkwabyu vuxokoxoko lebyi kumiweke ku pfuneta eka policy and practice.

MULTISECTORAL STAKEHOLDERS

Ku antswisa ku hlanganyela na ku teka magoza ya ku seketela community ekaku lulamisa swi phiqo swinga fikeleliwa hi ku andzelela maendlelo lawa ya nga vekiwa ka manual lowu. Stakeholders va nga rhambiwa ku suka ek a provincial and district DoH including PHC, MCHWYN, HAST and NCD programmes. As can representatives from the Department of Cooperative Governance and Traditional Affairs (COGTA), Department for Social Development (DSD), Department of Basic Education (DBE) and Department of Culture, Sport and Recreation (DCSR). Key local stakeholders include clinic committee, ward committee, Local Municipality and the sub-district.

CONNECTING TO THE HEALTH SYSTEM

Ku seketela ku pulana loku ku endliwaka hi PHC hiku tirhisa vumbhoni lebyi kumiweke eka community lebyi fambisanaka na kutikeriwa loko swita eka mavabyi, na ntokoto lowu va ngana wona hiku hanya na ku tikeriwa koloko, xikan'we na magoza lawa va lavaka kuya teka ku lulamisa ku tikeriwa loku xikombiso, clinic committee meetings, sub-district PHC review meetings, or district health management team (DHMT) meetings, community groups supported by CHWs can plan for engagement, including convening smaller sub units planning and review teams, and negotiating access to programmes (e.g., maternal, child, women's and youth health), and convene sub-sets of management groups to support with priority setting, review and feedback as appropriate.

REFERENCE LIST

Cavestro L (2003) Participatory rural appraisal concepts, methodologies and techniques

https://liberiafti.files.wordpress.com/2013/08/cavestro_participatory-rural-appraisal-concepts-methodologies-techniques.pdf

Cleary S, du Toit, A, Scott V, Gilson L (2018) Enabling relational leadership in primary healthcare settings: lessons from the DIALHS collaboration. Health Policy and Planning, 33: ii65-ii74.

https://researchonline.lshtm.ac.uk/id/eprint/4648712/1/Cleary_etal_2018_Enabling_relational_leadership.pdf

Convention on Wetlands (2008) A guide to participatory action planning and techniques for facilitating groups. Ramsar CEPA.

https://www.yumpu.com/en/document/read/25662605/a-guide-to-participatory-action-planning-and-techniques-ramsar-

JHPIEGO (2020) Gender-transformative leadership: a participatory toolkit for health workers, facilitator guide. JHPIEGO, Maryland, USA.

https://www.jhpiego.org/wp-content/uploads/2020/07/GTL-Full-Toolkit.pdf

Loewenson R, Kaim B, Mbuyita S, Makemba A (2006), Organizing people's power for health: participatory methods for a people centred health system, EQUINET, Harare, Zimbabwe.

https://www.equinetafrica.org/sites/default/files/uploads/documents/EQUINET_PRA_toolkit_for_web.pdf

Loewenson R, Laurell AC, Hogstedt C, D'Ambruoso L, Shroff Z (2014) Participatory action research in health systems: a methods reader/ TARSC, AHPSR, WHO, IDRC Canada, EQUINET, Harare, Zimbabwe.

https://equinetafrica.org/sites/default/files/uploads/documents/PAR_Methods_Reader2014_for_web.pdf

Loewenson R, Simpson S, Dudding R, Obando F, Beznec P (2021) Making change visible: evaluating evidence to advance social participation in health, an implementer's resource. TARSC.

https://www.tarsc.org/publications/documents/MCV%20Implementers%20Resource%202021%20for%20web.pdf

Oladeinde O, Mabetha D, Twine R, Hove J, Van der Merwe M, Byass P, Witter S, Kahn K, D'Ambruoso L (2020) Building cooperative learning to address alcohol and other drug abuse in Mpumalanga, South Africa: a participatory action research process. Global Health Action, 13:1, 1726722.

https://www.tandfonline.com/doi/full/10.1080/16549716.2020.1726722

Photovoice (2015)

https://photovoice.org/

WWW.VAPAR.ORG

This program is supported by the Health Systems Research Initiative from Department for International Development (DFID)/ Medical Research Council (MRC)/ Wellcome Trust/ Economic and Social Research Council (ESRC) (MR/N005597/1, MR/P014844/1) and with support from the University of Aberdeen Grand Challenges Research Fund (GCRF), Scottish Funding Council (SFC). The work is a collaboration between the MRC/Wits Rural Public Health and Health Transitions Research Unit (Agincourt), School of Public Health, Faculty of Health Sciences, University of the Witwatersrand, Johannesburg, South Africa, Directorate for Maternal, Child, Women and Youth Health and Nutrition, Mpumalanga Department of Health, South Africa, Queen Margaret University, Edinburgh, Scotland UK and the University of Aberdeen, Scotland UK

Permissions have been secured for the reproduction of all images

vapar

WWW.VAPAR.ORG

Mpumalanga Health Policy and Systems Learning Platform

Community Health Workers Community Mobilisation TRAINING MANUAL

Verbal Autopsy with Participatory Action Research (VAPAR)
